# Combinatorial treatment with natural compounds in prostate cancer inhibits prostate tumor growth and leads to key modulations of cancer cell metabolism

**DOI:** 10.1038/s41698-017-0024-z

**Published:** 2017-06-05

**Authors:** Alessia Lodi, Achinto Saha, Xiyuan Lu, Bo Wang, Enrique Sentandreu, Meghan Collins, Mikhail G. Kolonin, John DiGiovanni, Stefano Tiziani

**Affiliations:** 1 Department of Nutritional Sciences, The University of Texas at Austin, Austin, TX USA; 2 Division of Pharmacology and Toxicology, College of Pharmacy, The University of Texas at Austin, Austin, TX USA; 3 Dell Pediatric Research Institute, The University of Texas at Austin, Austin, TX USA; 4 The Brown Foundation Institute of Molecular Medicine, University of Texas Health Science Center at Houston, Houston, TX USA

## Abstract

High-throughput screening of a natural compound library was performed to identify the most efficacious combinatorial treatment on prostate cancer. Ursolic acid, curcumin and resveratrol were selected for further analyses and administered in vivo via the diet, either alone or in combination, in a mouse allograft model of prostate cancer. All possible combinations of these natural compounds produced synergistic effects on tumor size and weight, as predicted in the screens. A subsequent untargeted metabolomics and metabolic flux analysis using isotopically labeled glutamine indicated that the compound combinations modulated glutamine metabolism. In addition, ASCT2 levels and STAT3, mTORC1 and AMPK activity were modulated to a greater extent by the combinations compared to the individual compounds. Overall, this approach can be useful for identifying synergistic combinations of natural compounds for chemopreventive and therapeutic interventions.

## Prostate cancer: Combination of natural compounds limits tumor growth

Combinations of two molecules found naturally in edible plants synergistically help reduce tumor growth in a mouse model of prostate cancer. Stefano Tiziani and John DiGiovanni from the University of Texas at Austin, and colleagues screened a library of 142 natural compounds for the effects of each molecule, alone or in combination, on the viability of cells from mouse and human prostate cancer cell lines. Amongst other promising combinations, the researchers identified ursolic acid and curcumin as the most promising combination for inhibiting tumor growth. (These compounds are found naturally in apple peels and turmeric, respectively.) In mice with implanted prostate tumors, the two compounds synergistically reduced tumor volume and weight, while in cell culture the researchers showed that the compound-combination strategy modulated metabolism of a critical amino acid and other cell signaling pathways.

## Introduction

According to the World Health Organization, one-third of all cancer deaths are preventable through an increased consumption of natural compounds able to modulate key molecular signaling cascades that ultimately inhibit cancer cell proliferation and induce apoptosis.^1–5^ A number of dietary phytochemicals, including curcumin (CUR), ursolic acid (UA), epigallocatechin-3-gallate (or EGCG), resveratrol (RES), sulforaphane and 6-shogaol have shown potential chemopreventive effects in vitro and in vivo in either animal models or in clinical studies on several cancers,^5–9^ including prostate cancer (PCa).^1, 5, 10–15^ These bioactive compounds target inflammatory signaling pathways including Stat3 and NFκB in addition to other signaling pathways associated with cancer development and progression.^7, 8, 11, 16–19^ These studies have led to an increasing enthusiasm in developing novel strategies for cancer prevention and treatment.^5–9^ Thus, a pressing need has arisen to identify novel bioactive phytochemicals and to understand their therapeutic role and mechanisms of action.

The systematic identification of effective bioactive agents is very challenging, partly due to the low specificity of phytochemicals. In turn, their low toxicity and capability of inhibiting multiple pathways represents a resourceful long-term strategy for chemoprevention or treatment of cancer. For instance, the ability of these compounds to target multiple pathways might be advantageous to limit compensatory signaling feedback loops and cross-talk between cellular pathways and between different cell types inside the tumor microenvironment. The vast molecular diversity offered by natural compound libraries represents an invaluable resource for identification of synergistic combinatorial treatments. However, the systematic search of synergistic combinations is hampered by the many possible combinations, even for combinations involving only two compounds within a modest pool of candidates. For example, a single dose (and single replicate) screening of a chemical library of 100 compounds in combinations of two agents, would require testing 4950 combinations. To avoid the extensive experimental exploration of all the possible compound combinations, several methods have been developed aimed at predicting synergistic combinations. However, most of these methods focused on the identification of targeted inhibitors for a single enzyme rather than on monitoring the global response of cancer cells.

Metabolomics, an emerging field of biomedical and nutrigenomics research,^20–22^ entails the measurement of a comprehensive pool of small molecules, called the metabolome, in biological samples.^23–25^ Besides being nutrients essential for cell growth, in the context of cancer, metabolites represent sensitive markers of major alterations in cancer cell metabolism and contribute to oncogenic signaling. Lipid metabolism alteration is considered as another hallmark of cancer cells since lipid metabolizing enzymes are directly regulated by the activity of oncogenic signals. Moreover, fluctuations in local metabolite concentrations,^26–30^ especially glucose, fatty acids, and amino acids, influence the efficacy of chemotherapy in several human cancers including prostate.^31, 32^ The wealth of information obtained from the multivariate metabolic readout offers an unprecedented opportunity to investigate the metabolic consequences of the administration of natural compound combinations and thereby identify synergies in their chemopreventive activity, as well as their contribution to improve treatment outcome when administered in combination with current standard of care chemotherapeutics.

In this study, a high-throughput screening approach was used to screen a natural compound library (NCL) and evaluate the efficacy of phytochemicals, when administered alone and in combination of two compounds using murine and human cell lines. The effect of the most promising compounds (UA, CUR and RES) were tested in vivo in a murine allograft model of PCa, as individual and combination treatments. All the natural compound combinations resulted in synergistic effects on tumor volume and weight. Therefore, we further analyzed the molecular effects of the compounds (administered alone and in combination) in in vitro models of PCa using an untargeted metabolomics approach combining magnetic resonance spectroscopy (MRS) and mass spectrometry (MS). A number of metabolic pathways were affected by the synergistic combinatorial treatments and allowed discrimination of those which were unequivocally driven by the individual compounds. Moreover, metabolic flux analysis using isotopically labeled glutamine indicated that the combination of UA with either CUR or RES led to a blockade of glutamine uptake by the cancer cells possibly contributing to the efficacy of these combinations in hindering PCa growth. Collectively, the approach used in this paper demonstrates that the metabolic response induced by single agent screening alone may guide the development of novel combinatorial treatments from in vitro to in vivo models and could potentially be translated into human studies.

## Results

### Screening of NCL

**Fig. 1:**
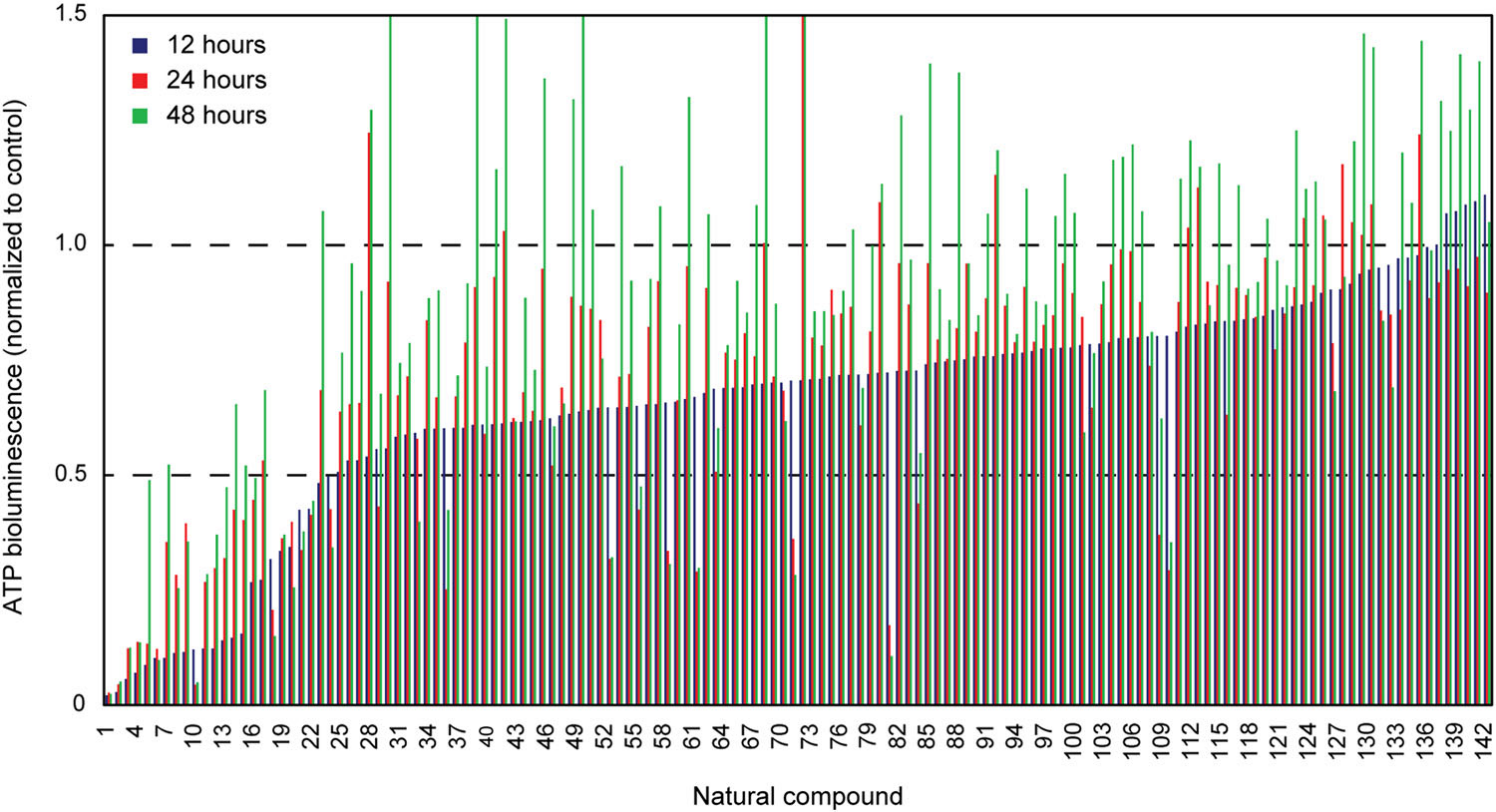
Screening of a natural compound library. HMVP2 cell viability following treatment with a library of natural compounds was screened using the ATP bioluminescence assay. HMVP2 cells were treated for 12 (*blue*), 24 (*red*) and 48 h (*green*) with a library of natural compounds (single agent) at the dose of 5, 10 or 20 µM (only the latter dose is shown in the figure). Natural compounds are sorted according to increasing ATP bioluminescence values of the 12 h time-point (list of compounds is included in Supplementary Table 1). Relative ATP levels (ATP level in treated samples normalized by ATP level of untreated samples) as averages of four replicates are shown

The effect of a library of 142 natural compounds (listed in Supplementary Table 1) on cell viability (based on the adenosine triphosphate, ATP, bioluminescence assay) was screened in the HMVP2 mouse PCa cell line.^33, 34^ HMVP2 cells were screened at three different concentrations (5, 10 and 20 µM) and three time points (12, 24 and 48 h). Z-factors were calculated to evaluate cell response (based on ATP suppression) following exposure to the natural compounds. A Z-factor value greater than 0.5 was considered a very good response. The 20 µM treatment resulted in the highest number of compounds with Z-factors greater than 0.5 and ATP values lower than 0.5 (indicating good suppression) and was therefore selected as the best dose for the selection of the top hits (Fig. 1, ATP only). ATP and the derived Z-factors for all treatments and all doses are included in Supplementary Table 1. ATP suppression in treated HMVP2 cells varied greatly, with the majority of the screened natural compounds inducing a moderate drop in ATP concentration to >50% of control (Fig. 1). Notably, after 12 h of treatment at the 20 µM dose, the majority of the natural compounds under study induced a greater suppression of ATP levels compared to untreated (solvent control) samples than longer treatments at the same dose (Fig. 1). Moreover, the top hits (selected based on the highest Z-factor) were very consistent regardless of the treatment dose (Supplementary Table 1) and include shikonin, dioscin, amygdalin, UA, and oridonin (only for 24 and 48 h of treatment). Shikonin and dioscin were highly effective at all time points and all doses; however, no differences in ATP suppression levels were observed under the experimental conditions for these two compounds (even at lower doses of 5 and 10 µM, as shown in Supplementary Table 1). These results are likely due to the concentrations being too high. Therefore, shikonin and dioscin were not granted further investigation in the metabolic analysis and will be further characterized to determine a more appropriate concentration range. In spite of the high Z-factor values, amygdalin was not selected as a top hit in this study due to the known ineffectiveness as a cancer treatment.^35^ UA was selected as the top hit for further combinatorial treatment studies, as this compound overall resulted in better outcomes than oridonin.

We next performed the screen in HMVP2 cells treated with UA (top hit from the individual agent screen) in combination with all the agents screened as individual treatments (both agents used at 20 µM for 12, 24 and 48 h). In addition to the HMVP2 cell line, we also performed the screen with the combined treatments in 3 androgen-independent, human PCa cell lines, DU145, PC3 and C4-2B (Supplementary Table 3). The experimental ATP-suppression values for the combined treatments are included in Table 1 (treatments inducing the top 20 highest ATP-suppression levels). In HMVP2 cells, the combination of UA with either corosolic acid, aesculin or CUR led to the highest level of ATP suppression (besides the combinations with shikonin and dioscin, excluded for the same considerations mentioned previously for the individual treatment screen). Corosolic acid is structurally analogous to UA and was therefore deemed suboptimal for further studies on the effect of the combined treatment. CUR and aesculin led to comparable ATP suppression results in HMVP2 cells, however, CUR showed greatly improved ATP suppression in DU145 and PC3 cells and similar ATP suppression in C4-2B cells compared to aesculin (Supplementary Table 3). Therefore, CUR was selected as top hit for combination treatment with UA. In addition to CUR, we also included in our metabolic studies the combination of UA with another natural compound, RES, which in our primary screen showed little to no ATP suppression (average control-normalized ATP levels between 0.77 and 1.72 in mouse and human PCa cell lines) when administered as a single agent, and a greatly improved ATP suppression following combined administration with UA. The combination of CUR and RES was also included for comparison. We also compared the effect on cell survival (using the MTT assay) of the combination of UA with either CUR or RES to a variety of standard care of therapeutic agents used for the treatment of castration-resistant prostate cancer including docetaxel, enzalutamide and abiraterone acetate in mouse HMVP-2 as well as human (PC3 and LNCaP) PCa cell lines. Both combinations of natural compounds showed at least comparable suppression of cell growth/survival (Supplementary Fig. 1) to the standard therapeutic agents.

**Table 1:** Cell viability modulation following combined treatment with UA and the NCL compounds

12 h		24 h		48 h	
Shikonin	0.007	Shikonin	0.008	Shikonin	0.008
Dioscin (Collettiside III)	0.009	Oridonin (Isodonol)	0.015	Oridonin (Isodonol)	0.015
Aesculin (Esculin)	0.022	Dioscin (Collettiside III)	0.015	Dioscin (Collettiside III)	0.025
Amygdalin	0.025	Ursolic acid (Malol)	0.031	Ursolic acid (Malol)	0.030
Corosolic acid (CA)	0.030	Corosolic acid (CA)	0.035	Corosolic acid (CA)	0.036
Curcumin	0.033	Amygdalin	0.045	Azomycin (2-Nitroimidazole)	0.040
Maslinic acid (MA)	0.037	Curcumin	0.046	Cyclocytidine HCl	0.040
Gossypol	0.037	Aesculin (Esculin)	0.053	Aesculin (Esculin)	0.045
Oleanolic Acid (Caryophyllin)	0.039	(+)-Usniacin (D-Usnic acid)	0.054	Tanshinone I	0.049
(+)-Usniacin (D-Usnic acid)	0.039	Oleanolic Acid (Caryophyllin)	0.061	(−)-Epigallocatechin gallate	0.049
Biochanin A (4-Methylgenistein)	0.041	Cyclocytidine HCl	0.061	(+)-Usniacin (D-Usnic acid)	0.050
Asiatic acid	0.041	Cryptotanshinone	0.062	Asiatic acid	0.056
Tetrandrine (Fanchinine)	0.044	Honokiol	0.065	Sclareol	0.061
4-Methylumbelliferone (4-MU)	0.047	Azomycin (2-Nitroimidazole)	0.074	Curcumin	0.061
Honokiol	0.047	(−)-Epigallocatechin gallate	0.076	Chrysin	0.062
Oridonin (Isodonol)	0.048	Cyclosporin A (Cyclosporine A)	0.076	Gossypol	0.062
Indirubin	0.049	Magnolol	0.080	3-Indolebutyric acid (IBA)	0.066
Magnolol	0.051	Tanshinone I	0.087	Hematoxylin (Hydroxybrazilin)	0.073
Sclareol	0.051	Gynostemma Extract	0.089	Arbutin (Uva, p-Arbutin)	0.074
Epi-Maslinic acid (EMA)	0.053	Baicalein	0.090	Cinchonidine	0.076

ATP bioluminescence values (mean value, normalized to control) in HMVP2 cells treated with the combination of UA and each of the compounds in the NCL, both at 20 µM and for 12, 24 or 48 h. At each time-point, values are sorted by increasing ATP and only the first 20 natural compounds are listed (complete results are included in Supplementary Table 2).

In addition to screening for modulation of cell viability (measured via ATP bioluminescence) following combined treatment with natural compounds, we also monitored generation of reactive oxygen species (ROS) in all the mouse and human PCa cell lines. Notably, screening of ROS levels indicated that UA and RES, administered either as single agents or in combination either decreased or did not affect the ROS levels (compared to control). In contrast, CUR alone induced an increase in ROS in most of the cell lines (not significant in PC3), which was suppressed by the combination with UA (Table 2).

**Table 2:** Induction of ROS in treated murine and human PCa cells

	CUR		RES		UA
	Single agent	Combination with UA	Single agent	Combination with UA	Single agent
HMVP-2	**1.67** ± **0.16**	**0.60** ± **0.03**	0.86 ± 0.15	**0.77** ± **0.09**	**0.81** ± **0.09**
DU145	**1.21** ± **0.12**	**0.47** ± **0.02**	0.79 ± 0.14	**0.64** ± **0.08**	**0.42** ± **0.04**
PC3	1.03 ± 0.20	**0.53** ± **0.09**	0.88 ± 0.11	0.86 ± 0.16	**0.65** ± **0.16**
C4-2B	**1.53** ± **0.28**	1.17 ± 0.19	**0.68** ± **0.15**	0.97 ± 0.29	1.02 ± 0.11

ROS levels (treatment normalized to control values) measured in mouse (HMVP2) and human PCa (DU145, PC3 and C4-2B) cell lines following treatment with resveratrol, curcumin (either as single agent or in combination with ursolic acid) and ursolic acid (all at 20 µM for 12 h). Values shown are mean ± standard deviation. Values in bold are significant (*p* < 0.05) according to student’s *t*-test.

### In-vivo effects of UA, CUR and RES administered alone or in combination

**Fig. 2:**
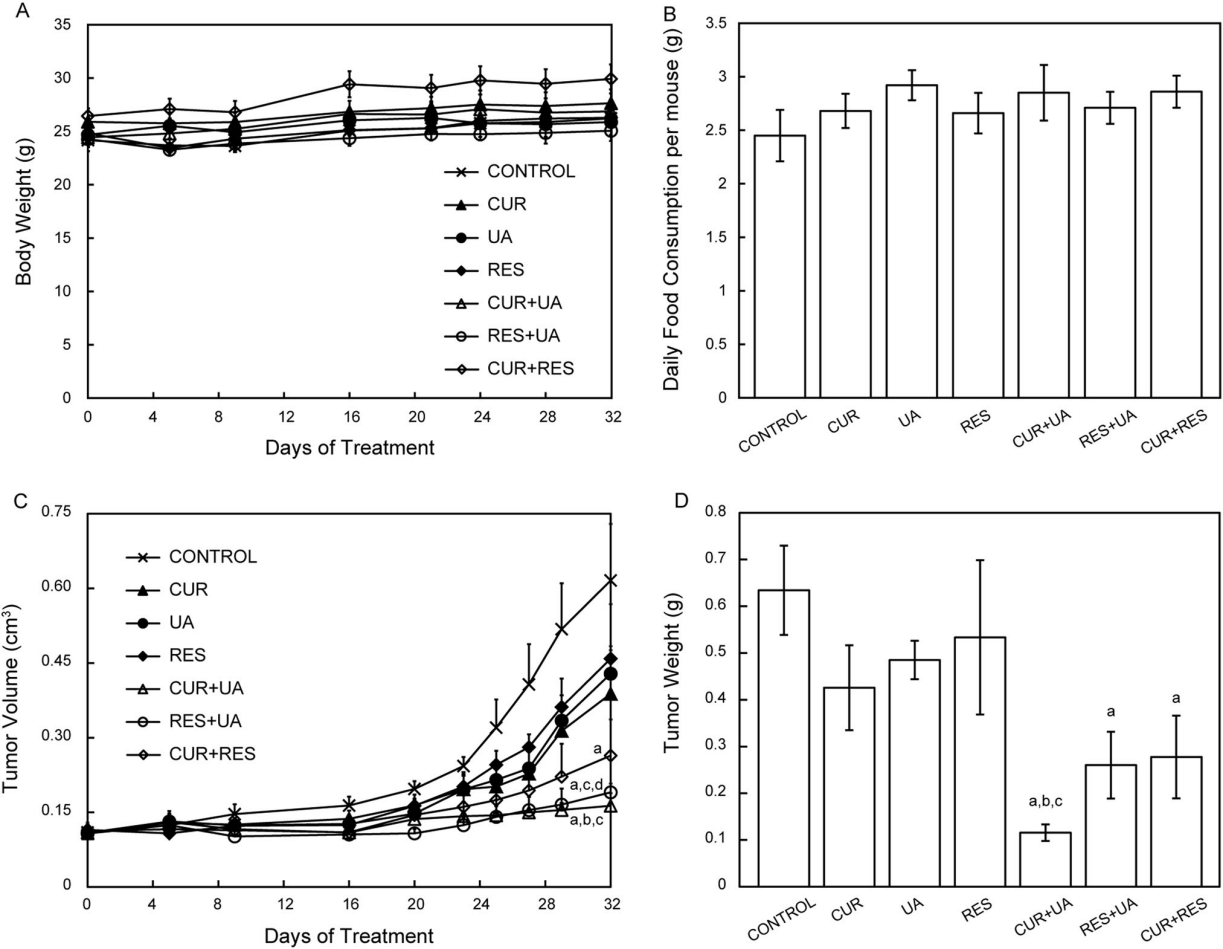
In vivo effect on HMVP2 tumor growth of treatment with CUR, UA, RES and their combinations. HMVP2 cell spheroids were injected subcutaneously into the flank of male FVB/N mice. Mice were fed ad libitum with semipurified AIN76A-based diet containing CUR, UA, RES or their combinations of two natural compounds. Body weight (**a**), food consumption (**b**), tumor volume (**c**) and tumor weight (**d**) are shown as mean ± SEM. One-way ANOVA with significance at *p* < 0.05 was used. Statistical significance is shown as different from control (**a**), CUR (**b**), UA (**c**) and RES (**d**)

After selecting the top-hit compounds of interest from the above screens the in vivo effects of the single or combined treatments with the selected natural compounds were evaluated on growth of tumors derived from HMVP2 spheroids in a mouse allograft model. HMVP2 cells were grown into spheroids, injected subcutaneously into the flank of male FVB/N mice and allowed to grow for 13 days post-injection before starting the treatment. All compounds were given in the diet. Tumor volume was monitored starting on the 1st day of treatment and continued until mice were sacrificed on day 32 of treatment. Moreover, tumors were weighed at the end of treatment (day 32). Notably, the addition of the natural compounds into the formulation of the animals’ diets did not induce changes in either the animals’ body weight (Fig. 2a) or their daily food consumption (Fig. 2b). Treatments administered as single agents induced modest, but not statistically significant, decreases in both tumor volume and weight (Fig. 2c and d) as compared to mice on the control diet. In contrast, all of the combinations resulted in significantly smaller tumors (both volume and weight) than mice on the control diet. Furthermore, the combination of UA + CUR also resulted in significantly smaller tumors (both volume and weight) than those obtained following administration of UA or CUR as single-agent treatment. Thus, overall the combination of UA + CUR appeared to be the most effective at inhibiting tumor growth, although all three combinations produced significant combinatorial effects. We also compared the effect of the combination of natural compounds on in vivo tumor growth with docetaxel. At the doses used for this experiment, docetaxel did not produce any significant effects in the HMVP2 allograft model or in HMVP2 cells treated in vitro (Supplementary Fig. 2).

To further evaluate the effectiveness of the natural compounds as combined treatments compared to the individual agents, we used the Bliss method^36^ to investigate whether any of the treatments resulted in synergistic effects. Using this method, all the combined treatments produced synergistic effects on tumor volume and tumor weight, as the predicted combined affected fraction was lower than the experimental value in all cases (Table 3; positive Bliss indices in all instances for tumor volume and weight).

**Table 3:** Evaluation of synergistic effects by combined treatments in vivo and in vitro

	Tumor volume^a^	BLISS index	Tumor weight^a^	BLISS index	Distance	BLISS index
CUR	0.63		0.67		0.27	
UA	0.70		0.76		0.34	
RES	0.74		0.84		0.23	
CUR + UA	0.27	0.17	0.44	0.13	0.65	0.13
RES + UA	0.31	0.21	0.41	0.23	0.58	0.09
CUR + RES	0.43	0.04	0.18	0.33	0.42	−0.02

Average tumor volume and weight (normalized to control group) in mice treated with single or combined agents and the associated Bliss index (calculated as difference between the experimental and the predicted combined affected fraction) for the effect of the combined treatments on tumor volume and weight. Mean experimental Euclidean distance between treatment groups in the PCA scores plot (Fig. 4) and associated Bliss index.

^a^ normalized to control

### Effect of treatment with the natural compounds on PCa cell metabolism

Given the encouraging outcome of the combined treatments observed in the in vivo study, the molecular basis for the efficacy of these natural compounds was further evaluated by performing a study of cell metabolism. HMVP2 cells were treated with the individual natural compounds selected above and their combinations. Intracellular and extracellular polar metabolites were profiled using a combination of ultra-high pressure liquid chromatography (UHPLC)-MS and MRS. Intracellular lipid metabolites were also analyzed using UHPLC-MS.

#### Polar metabolites

**Fig. 3:**
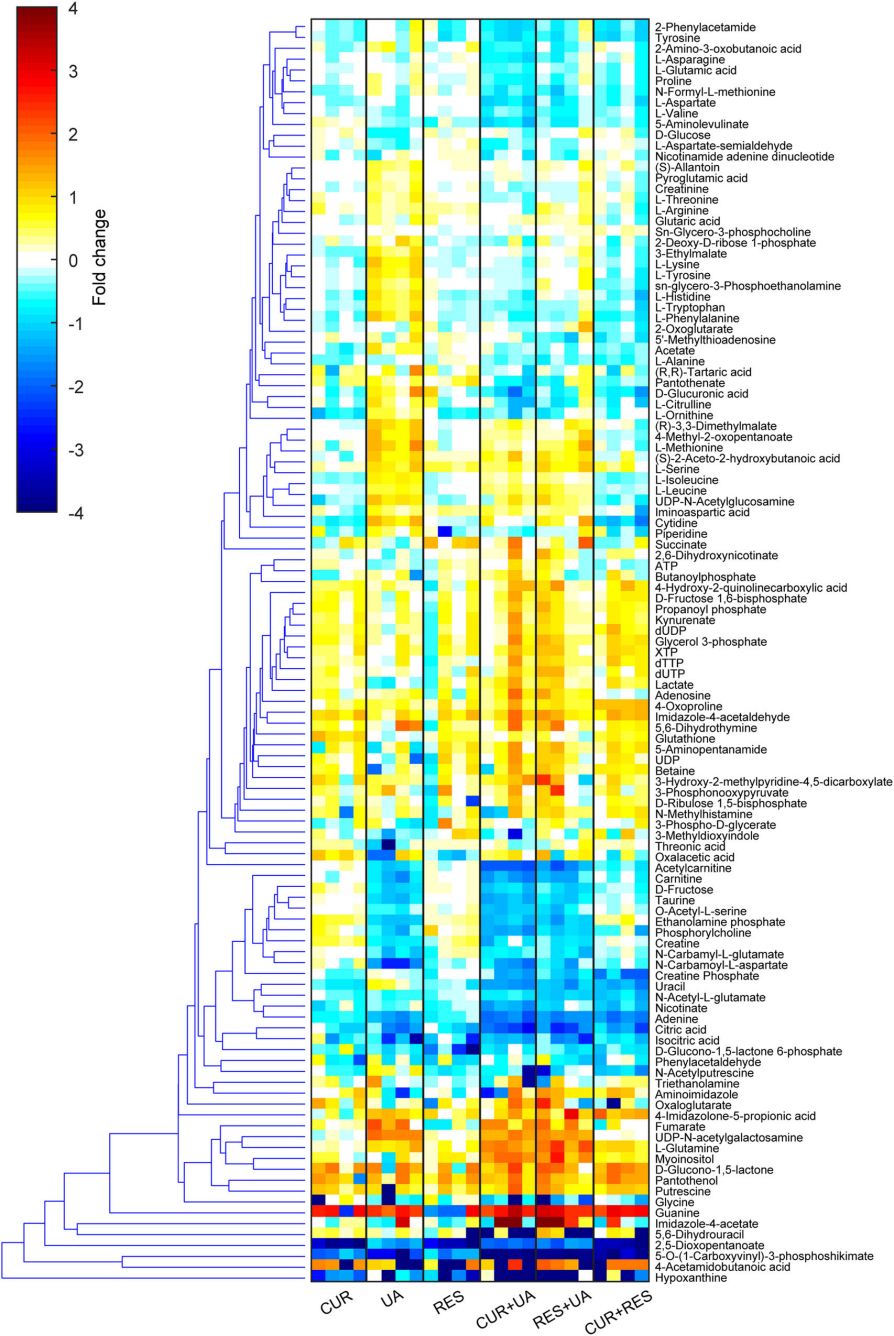
Metabolite level modulation in HMVP2 cells following treatment with CUR, UA, RES or their combinations. HMVP2 cells were treated for 12 h with 20 μM CUR, UA, RES or their combinations of two compounds. Fold change (compared to untreated samples) of polar intracellular metabolite levels measured by UHPLC-MS and MRS. Hierarchical clustering was performed using the Euclidean distance and average linkage clustering methods on the intracellular levels (normalized to control) of all the identified metabolites

The UHPLC-MS analysis of the polar intracellular metabolites yielded a total of approximately 600 valid features from which 103 metabolites (included in the Kyoto Encyclopedia of Genes and Genomes (KEGG) database) were identified. Thirty-nine metabolites were identified using the MRS analysis of which 14 were unique to the MRS analysis (not identified using the MS analysis). Fold change (normalized to control) of the metabolite levels in the treated cells are included in Fig. 3.

**Fig. 4:**
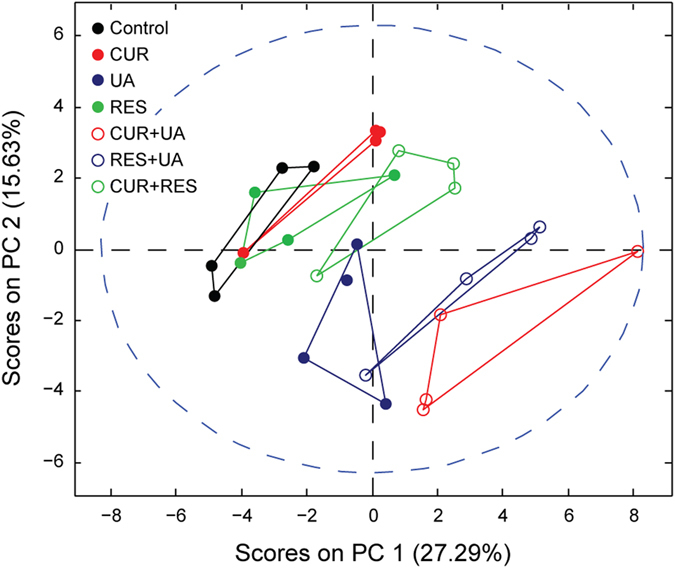
Treatment-induced metabolic changes in HMVP2 cells. HMVP2 cells were treated with CUR, UA and RES or their combinations of two compounds. The PCA scores plot (PC1 vs. PC2) was obtained from the unsupervised multivariate analysis of the metabolic profile data (polar fraction) acquired using UHPLC-MS and MRS

In order to visualize and characterize the global effect on cell metabolism of the different treatments, we performed principal component analysis (PCA), an unbiased and unsupervised multivariate method of analysis, on all the identified polar metabolites. The PCA scores plot (Fig. 4) clearly indicates that, among the single agent treatments, UA had the strongest effect on cell metabolism. Also CUR induced a relevant metabolic change while the effect of RES on HMVP2 cell metabolism was relatively small. To further evaluate the effect of the compound combinations on cell metabolism we calculated the Euclidean distance between all treatment groups and control in the 2-dimensional (PC1 vs. PC2) PCA scores plot (Table 3). As expected, given the outcome of the single agent treatments, the combination CUR + UA affected cell metabolism the most as shown by the distance from the control group in the PCA scores plot. In order to evaluate whether the effect of phytochemical combinations on cell metabolism were synergistic or at least additive we adapted the Bliss-independence theory^36^ to the normalized distance (Euclidean distance between treatment groups and control, normalized to the maximum distance between all points in the PCA scores plot) and estimated the predicted distance (Table 3). For the combinations of UA with either CUR or RES, the predicted combined affected fraction had values lower than the experimental ones. Therefore, the combinations of UA with the other natural compounds have synergistic effects on cell metabolism according to the Bliss independence based calculation.

**Fig. 5:**
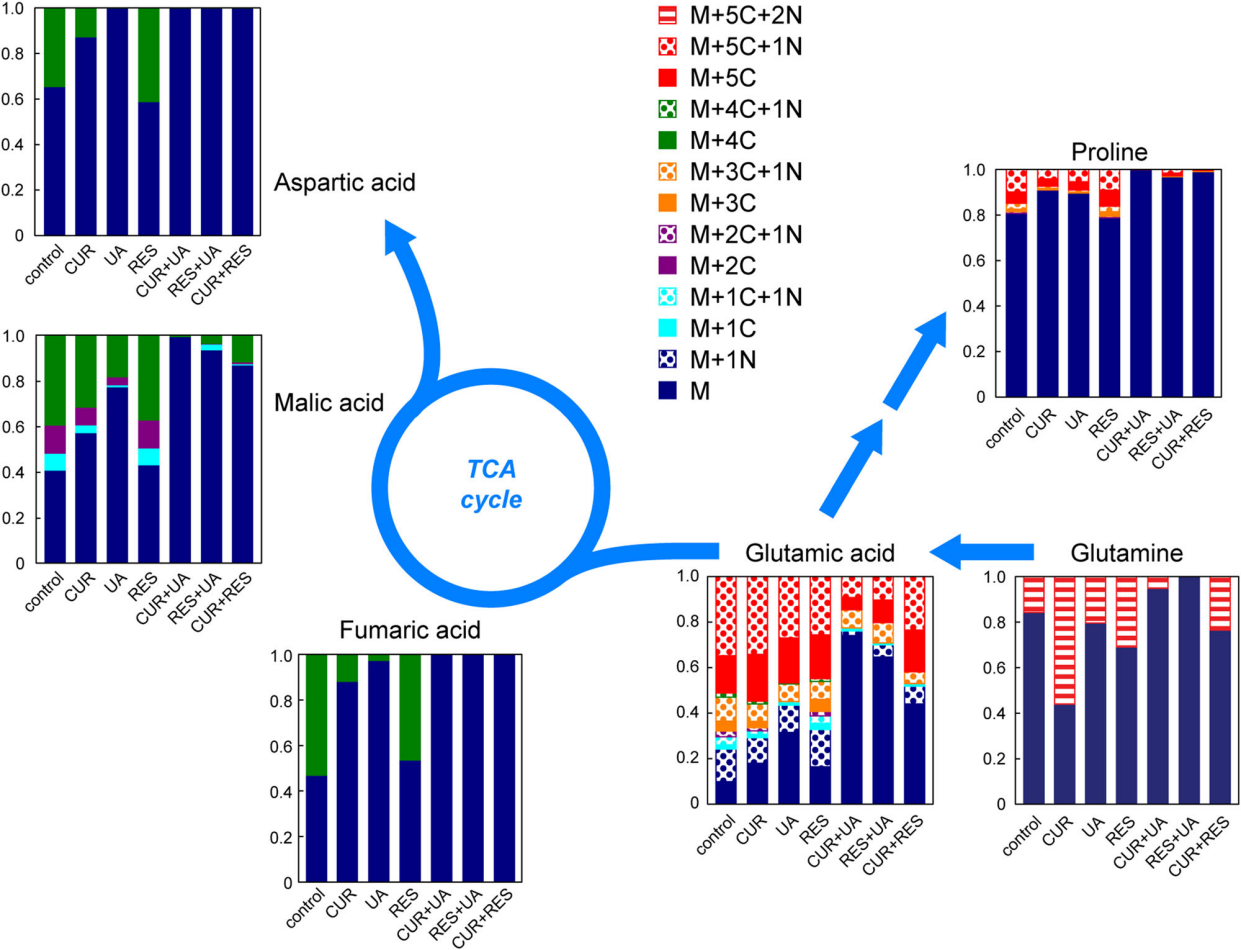
Treatment-induced changes in HMVP2 intracellular glutamine flux. HMVP2 cells were treated with CUR, UA, RES or their combinations of two compounds and concurrently cultured in medium containing ^13^C_5_,^15^N_2_-glutamine (glutamine in the growth medium completely replaced with isotopically labeled glutamine). Schematic representation of the flux of isotopically labeled glutamine through the TCA cycle, and the aspartate and proline metabolic pathways. UHPLC-MS data were acquired and metabolite enrichment is shown as the fraction of enrichment of the total metabolite pool (average of three replicates)

The effect of the individual and combined treatments on specific metabolic pathways was also evaluated using metabolic pathway analysis (Table 4). In line with the results of the PCA, pathway analysis indicated that treatment with RES alone induced much smaller changes than the individual administration of CUR or UA. UA, which induced the most significant metabolic changes as an individual treatment, likely drives the changes for most metabolic pathways when administered in combination with CUR or RES. Notably, the administration of UA in combination with both CUR or RES greatly enhanced the modulation of a number of metabolic pathways, including the “Alanine, aspartate and glutamate metabolism” and the “tricarboxylic acid (TCA) cycle” (Table 4). To gain a better understanding of the specific molecular basis responsible for these metabolic changes we performed isotopic labeling of treated HMVP2 cells with ^13^C_5_,^15^N_2_-glutamine, as this nutrient is metabolized through the pathways of interest (according to the metabolic pathway analysis in Table 4). Intracellularly,^13^C_5_,^15^N_2_-glutamine (Fig. 5) was detected at low levels (as percentage of the total glutamine pool) in cells treated with UA in combination with either CUR (5.4% ± 3.1%) and RES (undetected). UA administration alone resulted in variable levels of intracellular ^13^C_5_,^15^N_2_-glutamine (13.4% ± 23.2%) that are not significantly different from those detected in the combined-treatment samples. The low levels of ^13^C_5_,^15^N_2_-glutamine resulting after the combined UA treatments could imply either that the uptake of glutamine from the extracellular space is reduced following UA-containing combined treatments or that glutamine is rapidly converted (more so than with the other treatments) to other metabolites following uptake. However, a very small flux of labeled carbons was observed in TCA cycle intermediates as well as other metabolic pathways (e.g. proline) in samples treated with UA-containing agent combinations, in line with the reduced glutamine uptake from the extracellular medium. Notably, the amount of labeled glutamine flux in UA-containing combined treatments is overall much lower than any of the individual agents administered alone. The analysis of metabolite levels in the extracellular space (in growth medium) indicated that glutamine consumption over the 12 h treatment period was comparable (no significant differences) for all treatments (data not shown). However, the relatively small amount of glutamine uptake over the short treatment period and the large initial glutamine levels in the medium might mask the changes in glutamine uptake resulting in the extracellular space.

**Table 4:** Metabolic pathway analysis in HMVP-2 cells

	Hits	Impact	CUR	RES	UA	CUR + RES	CUR + US	UA + RES
Alanine, aspartate and glutamate metabolism	10/24	0.76	0.190	0.815	0.110	0.051	0.001	0.017
Arginine and proline metabolism	14/44	0.54	0.073	0.399	0.002	0.035	0.001	0.007
Ascorbate and aldarate metabolism	2/9	0.40	0.190	0.399	0.216	0.007	0.003	0.037
Citrate cycle (TCA cycle)	6/20	0.33	0.421	0.605	0.161	0.202	0.008	0.031
D-Glutamine and D-glutamate metabolism	3/5	1.00	0.274	0.611	0.155	0.035	0.002	0.029
Glutathione metabolism	6/26	0.45	0.073	0.380	0.099	0.035	0.022	0.110
Glycine, serine and threonine metabolism	8/31	0.60	0.147	0.481	0.009	0.110	0.001	0.007
Glyoxylate and dicarboxylate metabolism	3/18	0.39	0.333	0.605	0.161	0.151	0.030	0.049
Histidine metabolism	7/15	0.52	0.179	0.605	0.017	0.024	0.003	0.015
Methane metabolism	2/9	0.40	0.469	0.380	0.013	0.110	0.035	0.017
Nicotinate and nicotinamide metabolism	2/13	0.21	0.190	0.126	0.408	0.035	0.008	0.079
Phenylalanine metabolism	4/11	0.54	0.277	0.493	0.027	0.051	0.021	0.626
Phenylalanine, tyrosine and tryptophan biosynthesis	2/4	1.00	0.190	0.399	0.003	0.043	0.016	0.439
Pyrimidine metabolism	11/41	0.27	0.077	0.481	0.018	0.032	0.010	0.010
Taurine and hypotaurine metabolism	1/8	0.43	0.244	0.704	0.001	0.100	0.000	0.005
Valine, leucine and isoleucine biosynthesis	4/11	1.00	0.081	0.611	0.000	0.004	0.002	0.001

Metabolic pathway analysis was performed on the identified polar metabolites. Pathways with impact greater than 0.2 are included. The column “Hits” includes the number of identified compounds over the total number of compounds in the specific pathway

#### Lipid metabolites

The results of the intracellular lipid analysis are included in Supplementary Results.

### Effect of treatment with the natural compounds on relevant cell signaling pathways

**Fig. 6:**
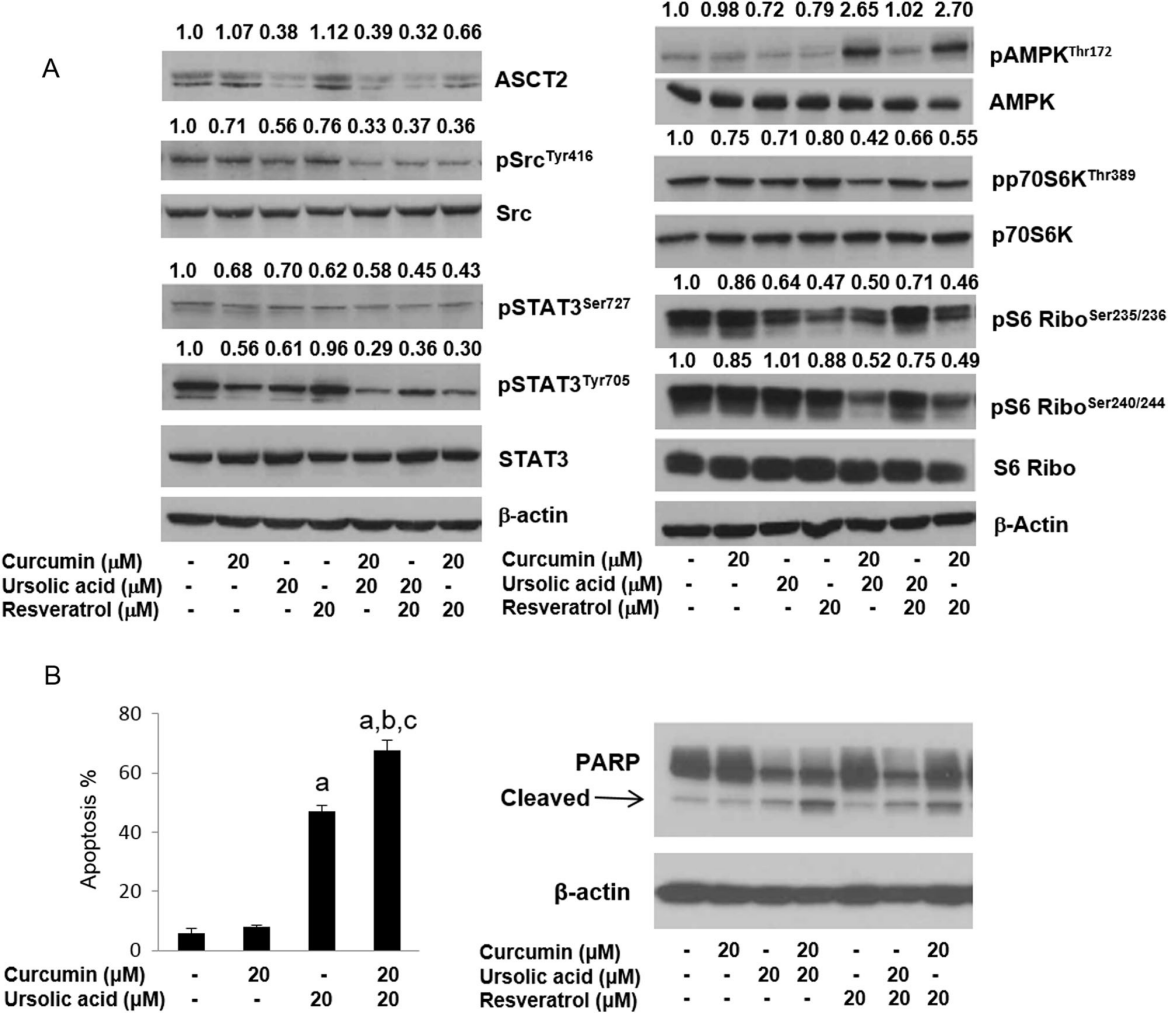
Effect of treatment on glutamine transport and relevant signaling pathways and apoptosis. **a** HMVP2 cells were treated for 2 h with 20 μM CUR, UA, RES or their combinations of two compounds. The levels of the glutamine transporter ASCT2, and phospho and total protein levels for Src, STAT3, AMPK, p70S6K and S6 were measured by Western blotting. Western blotting was performed two times with β-actin controls for each experiment. Numbers *above* blots indicate band intensities (normalized to control). **b** The percent of apoptotic cells (measured by Annexin V) and the levels of apoptotic markers (Western blotting) were probed after 24 h of treatment with the natural compounds. One-way ANOVA with significance at *p* < 0.05 was used. Statistical significance is shown as different from control (**a**), CUR (**b**) and UA (**c**)

Since the combination of CUR + UA and UA + RES decreased the uptake of glutamine and ASCT2 is the major glutamine transporter in the cancer cells, the level of ASCT2 after treatment with phytochemicals and their combinations was measured. As shown in Fig. 6, CUR and RES alone did not alter the ASCT2 in HMVP2 cells. However, UA, either alone or in combination with CUR or RES, decreased the protein level of ASCT2. To further understand the underlying mechanisms associated with the outcome of single or combined phytochemical intervention, the effects of treatment on several important cell signaling pathways involved in cancer, such as AMPK, mTORC1, Src and STAT3 signaling, were explored.As shown in Fig. 6, all the combination treatments decreased phosphorylation of STAT3 both at tyrosine705 and serine727 more than the reduction of phosphorylation resulting from any of the single agents alone. Src is a known upstream regulator of STAT3 signaling. All the combinations of these natural compounds also decreased phosphorylation of Src (Fig. 6), with CUR + UA inducing the largest effect. Next, the effect of the natural compound combinations on AMPK and mTORC1 signaling were examined. Both the combinations containing CUR (i.e., CUR + UA and CUR + RES) resulted in rapid activation of AMPK and a decrease in mTORC1 activity (as assessed by decrease in phosphorylation of p70S6K and downstream S6 ribosomal protein). The combination of UA + RES also increased AMPK phosphorylation and decreased mTORC1 activity at later time points (Fig. 6 and Supplementary Fig. 3). Since the combinations of UA with CUR or RES showed concurrent activation of AMPK and inhibition of mTORC1, we next examined AMPK and mTORC1 signaling with known regulators of these signaling pathways, such as metformin (activator of AMPK) and rapamycin (mTOR inhibitor) in HMVP2 and LNCaP cells. Consistent with our previous report,^37^ metformin-activated AMPK and rapamycin decreased mTORC1 signaling in both mouse and human PCa cell lines (Supplementary Fig. 4). In line with the HMVP-2 results, the combination treatments with natural compounds also decreased STAT3 phosphorylation in DU145 cells (Supplementary Fig. 5). Importantly, administration of the combinations of natural compounds was associated with increased apoptosis in both HMVP-2 cells (Fig. 6 or Supplementary Fig. 3) and DU145 cells (Supplementary Fig. 5).

## Discussion

In this study, we screened a library of natural compounds for efficacy on the survival of PCa cells. We performed a primary screen based on depletion of cellular ATP levels. Cellular ATP levels are critical for cell survival, and several reports have shown that reductions in cellular ATP levels can lead to apoptosis and other types of cell death in cancer cells, depending on the level of depletion.^38–42^ Based on cell viability (from ATP levels) of the primary screen, we selected the top hit natural compound (UA) and performed an additional secondary screen to investigate the effect of the administration of the top hit in combination with the natural compounds in the library. Notably, UA had previously been shown to deplete ATP in a glioblastoma cell line leading to necrotic cell death^43^ and to exert anticarcinogenic properties in several animal models of cancer.^18, 44–46^

For the secondary analysis, we screened both cellular ATP depletion levels and induction of ROS. Induction of ROS has been previously associated with reduced tumor cell growth for a number of tumor types.^47^ From the secondary screens, we identified two additional compounds of interest (CUR and RES) that induced marked reductions in cellular ATP levels when administered in combination with UA. CUR and RES have both been widely studied as chemopreventive agents.^7, 48, 49^ Clinical trials have been conducted with both CUR and RES administered as single agents for cancer chemoprevention.^50–52^ In particular, CUR has been studied in a number of clinical trials, either completed or ongoing, for a variety of cancers, including solid tumors.^53^ In line with the results of previous reports,^16^ CUR alone promoted increased ROS. However, all the other individual treatments (UA and RES), as well as the combination of both CUR and RES with UA either had no effect or induced drops in ROS levels depending on the PCa cell line. Therefore, the combined treatment efficacy observed in thisin vitro study is likely not determined by induction of ROS but has an alternative molecular origin. Before moving on to a more detailed investigation of the molecular mechanism of action of the selected natural compounds, we validated the effect of the combined treatments on tumor growth in a mouse allograft model of PCa. The in vivo studies provided further evidence that the selected natural compound combinations were highly effective in slowing growth of murine PCa cells. Notably, all the combinations of the selected natural compounds led to synergistic effects on tumor volume and weight (according to the Bliss index); in all cases, the combined administration of these natural compounds produced tumors of much smaller size than in untreated mice or mice receiving the individual compounds, with the combinations of UA with either CUR or RES yielding the best results.

Additional analysis of the treatment induced metabolic changes revealed a strong influence of both the combinations of UA either CUR or RES on glutamine/glutamate-related metabolic pathways. Metabolic analysis of the flux of isotopically labeled glutamine pointed to a decreased glutamine uptake from the extracellular space in cells treated with UA in combination with CUR or RES. Notably, the dependence on increased amino acid transport (i.e., increased expression of the amino acid transporters) for prostate cell growth has been previously reported, as well as the hindered PCa cell growth following inhibition of the ASCT2 glutamine transporter.^54, 55^ In our study, the levels of ASCT2 decreased following treatment with UA alone as well as its combinations with either CUR or RES. Moreover, the combination of CUR + RES also resulted in decreased levels of ASCT2 (although less dramatic than for the other combinations). Therefore, the modulation of ASCT2 alone did not fully explain our observation of glutamine metabolism nor the possible association with the improved in vivo outcome.

Recent studies reported that glutamine mediates oncogenic transformation in highly invasive ovarian cancer cells through STAT3 signaling.^56^ Given the decreased cellular uptake of glutamine resulting from the combined treatments with UA + CUR and UA + RES, we performed Western blotting to probe the level of phospho-STAT3. All three phytochemical combinations decreased the phosphorylation of Stat3 at both serine and tyrosine residues. Similarly, all three combinations also decreased phosphorylation of Src, in line with previous studies that also reported the decrease in Src phosphorylation in cells under conditions of both glutamine and glucose deprivation.^56^ Our additional mechanistic studies revealed a decrease in mTORC1 activity by the combination of phytochemicals. In this regard, Wang et al. showed that knockdown of ASCT2 by shRNA in PC-3 cells decreased the mTORC1 activity (measured by p70S6K phosphorylation) both in culture and in xenograft tumor tissues.^54^ Finally, as UA and the combinations decreased the cellular ATP level and depletion of cellular ATP level is associated with AMPK activation (reviewed in),^57^ we also measured the AMPK phosphorylation after treatment with the individual agents or their combinations. The combinations of CUR + UA and CUR + RES induced AMPK activation compared to untreated samples or treatment with the individual agents. Activation of AMPK might also be associated with the observed decrease in mTORC1 activity. Overall, as is expected for natural compounds, the outcome induced by the individual and, even more so, the combined treatments is due to complex effects entailing several cellular targets and pathways. While additional molecular targets are likely to further contribute to the beneficial effect of treatment with the selected natural compounds, the metabolic effect on glutamine metabolism and the associated effects on several key signaling pathways (STAT3/Src, mTORC1 and AMPK) likely contributed to the induction of apoptosis observed in HMVP2 cells in vitro and the synergistic inhibitory effects on tumor growth observed in vivo.

In conclusion, an initial screening approach has been developed to identify potential synergistic phytochemical combinations with chemopreventive/therapeutic efficacy for inhibiting growth of PCa and possibly other cancer cells. The ability of combinations to synergistically inhibit tumor growth was linked to synergistic changes in glutamine metabolism, the associated modulation of STAT3/Src, mTORC1 and AMPK signaling, and induction of apoptosis.

## Materials and methods

### Cell cultures

The HMVP2 murine PCa cell line derived from 1 year old HiMyc transgenic mice and NMVP cell line derived from FVB/N mice was cultured and spheroids generated as recently described.^34^ Human PCa cell lines, LNCaP, DU145, PC-3 and C4-2B were purchased from the American type culture collection (ATCC; Manassas, VA, USA). All cell lines were grown in 95% air and 5% CO_2_ at 37 °C. Both murine and human cell lines were maintained in RPMI-1640 medium (HyClone, Logan, UT, USA) and supplemented with 10% fetal bovine serum (HyClone) and 2 mM glutamine (Thermo Fisher Scientific, Waltham, MA). All the experiments were performed within 6–12 months of cell line authentication (using short tandem repeat analysis; PowerPlex 1.2 System, Promega, Madison, WI, USA) and mycoplasma testing (MycoAlert; Lonza, Rockland, ME, USA).

### High-throughput screening

A NCL of 142 phytochemicals (Supplementary Table 1) was screened on PCa cells. The NCL includes Selleck Chemicals’ NCL (Houston, TX, USA) and 11 additional natural compounds. Betulinic acid, caffeic acid phenylethyl ester, genistein, palmatine chloride, shikonin and RES were purchased from CaymanChemicals (Ann Arbor, MI, USA), whereas >98% pure corosolic acid, epi-maslinic acid, epi-UA, epi-corosolic and maslinic acid were prepared as recently described.^58, 59^ For all screens, cells in exponential proliferation were seeded in 384-well plates using a Viaflo Assist micro-plate dispenser (Integra Biosciences, Hudson, NH, USA) and allowed to adhere overnight. During the primary screening, HMVP2 cells were screened with the individual NCL compounds at 3 time-points (12, 24 or 48 h) and three doses (5, 10, 20 µM final concentration). A secondary screen was then performed on both the murine and human cell lines treated for 12 h with combinations of two compounds administered at the same concentration (20 µM final concentration). The NCL was dispensed into the seeded 384-well plates using the Viaflo-384 liquid-handling system (Integra Biosciences). To reduce bias, the treatments were administered based on a randomized list of well positions.

Two bioluminescence assays, CellTiter-Glo kit and ROS-Glo kit (both from Promega, Madison, WI), were used according to the manufacturer’s protocols to measure cell viability and ROS. Both ATP and ROS-based measurements were acquired on a SpectraMax M5e Series Multi-Mode Microplate Reader (Molecular Devices, Sunnyvale, CA) with 4 replicates per condition. Relative luminescence values were calculated as the luminescence value of the treated sample over that of solvent control cells within the same plate.

In addition, the NMVP and HMVP2 cell lines were seeded in 96-well plates and treated with docetaxel at 1, 10, 25, 50,100 and 250 nM for 12 h. Cell viability was investigated by performing the ATP bioluminescence assay.

### In vivo tumor growth

All animal husbandry and experiments were carried out in strict accordance to guidelines defined by the Association for Assessment and Accreditation of Laboratory Animal Care and approved by the institutional animal research committees at The University of Texas at Austin.

Spheroids generated from HMVP2 cells were injected subcutaneously into the flank of male FVB/N mice. Thirteen days after injection of spheroids, treatment with the natural compounds was started by switching animals to semipurified AIN76A-based diets containing 1.0% CUR, 0.2% UA, 0.5% RES or their combinations (all ad libitum). Body weight and food consumption were monitored weekly in all groups. Starting on day 1 of treatment, tumor volume was measured twice weekly using digital calipers. Treatment was continued for 32 days and the experiment was terminated 45 days after injection of the tumor cell spheroids. At the end of the treatment period, mice were killed, and tumors were excised and weighed. The effect on tumor weight and volume was also evaluated following treatment with docetaxel. Four days after the injection of spheroids, mice were treated weekly with docetaxel (20 mg/kg body weight with half of the drug volume injected intraperitoneally and half subcutaneously) for four consecutive weeks. Tumor volume was measured weekly and tumor weight was measured at the end of the study.

### HMVP2 cell extracts for metabolomics and isotopic labeling analysis

HMVP2 cells were treated for 12 h with either solvent control dimethyl sulfoxide or UA, CUR and RES administered alone or in combination. For the glutamine flux analysis, natural abundance glutamine in the medium was completely replaced with ^13^C_5_, ^15^N_2_-glutamine (Cambridge Isotopes Laboratories, Inc.). At the end of the 12-h treatment period, an aliquot of media was collected from each flask for extracellular analysis. Cells were washed twice with PBS, harvested and intracellular metabolites were extracted using a modified Bligh-Dyer procedure, as previously reported^60–63^ (as described in Supplementary Information).

### MRS-based metabolomics analysis

Polar samples for MRS analysis were resuspended in 45 µl of 0.1 M phosphate buffer in 90% D_2_O with 1 mM TSP, 0.05% NaN_3_, vortexed and centrifuged at 4 °C for 10 min. Thirty-five microliters of the supernatant were transferred into 1.7-mm tubes. One dimensional ^1^H MRS spectra were acquired and processed as previously described^29, 63, 64^ (as described in Supplementary Information).

### MS-based metabolomics analysis

Both polar and lipid analyses were analyzed on a Q Exactive Hybrid Quadrupole-Orbitrap Mass Spectrometer (Thermo Fisher Sci., Bremen, Germany). Sources for all solvents and reagents, and more detailed experimental procedures are included in Supplementary Information. Chromatographic separation was performed on a Thermo Scientific (Thermo Fisher Sci., San José, CA, USA) Accela UHPLC system equipped with a quaternary pump, vacuum degasser and an open autosampler with a temperature controller. MS analysis was carried out on a QEx active benchtop Orbitrap detector loading an electrospray (ESI) source simultaneously operating in fast negative/positive polarity switching ionization mode. For all sample types (media, and polar and apolar intracellular fractions), quality control samples were run once every five samples.

All raw MS datasets were processed using Sieve 2.2 (Thermo Scientific) and mined against an in-house database of accurate masses and retention times generated in our laboratory using the IROA 300, MS Metabolite Library of Standards (IROA Technologies, Bolton, MA). In addition, databases of accurate masses taken from the KEGG database^65^ and the Human Metabolome database^66^ were also mined. MS data were then combined to the MRS data for the subsequent post-processing, followed by univariate and multivariate statistical analyses.

### Treatment of HMVP2 cells and western blotting

HMVP2 cells were treated with the indicated concentrations of CUR, UA, RES or their combinations for the specified time (2, 6,and 24 h). Experimental procedures and the specific primary antibodies used are detailed in Supplementary Information. For all the Western blots shown, samples were derived from the same experiment and were processed in parallel. Molecular weight markers are shown in Supplementary Fig. 5.

### Statistical and pathway analyses and evaluation of synergy between natural compounds

For the screens, Z factor was calculated from ATP values to identify top-hit compounds as previously reported. Z-factor values between 0.5 and 1 were considered as excellent separation of groups; 0 ≤ Z ≤ 0.5 as moderate separation while *Z* = 0 as poor separation.

For the in vivo data, one way analysis of variance (ANOVA) was used with *p* < 0.05 considered significant.

PCA was carried out in PLS-Toolbox (Eigenvector Research, Manson, WA, USA). Hierarchical cluster analysis was performed using the Euclidean distance and average linkage clustering methods on the intracellular levels (normalized to control) of all the identified metabolites. The pathway analysis of the polar metabolites was carried out using MetaboAnalyst 3.0.^67^

To evaluate whether the combination of two natural compounds induced any synergistic effects compared to the individual effectiveness we used the Bliss Independence Model.^36^ This approach is based on the probabilistic concept of independence; it predicts the theoretical combined affected fraction C following treatment with two combined single agents with individual affected fractions A and B, according to the formula C = A + B – AB. The predicted combined affected fraction with values lower than the experimental one is considered synergistic.^36^

## Electronic supplementary material

Supplemental Information

Supplemental Figure Legend

Supplementary Figure 1

Supplementary Figure 2

Supplementary Figure 3

Supplementary Figure 4

Supplementary Figure 5

Supplementary Tables

## Electronic supplementary material

**Supplementary Information** accompanies the paper on the *npj Precision Oncology* website (doi:10.1038/s41698-017-0024-z).
