# Erratum: Combinatorial treatment with natural compounds in prostate cancer inhibits prostate tumor growth and leads to key modulations of cancer cell metabolism

**DOI:** 10.1038/s41698-017-0027-9

**Published:** 2017-09-04

**Authors:** Alessia Lodi, Achinto Saha, Xiyuan Lu, Bo Wang, Enrique Sentandreu, Meghan Collins, Mikhail G. Kolonin, John DiGiovanni, Stefano Tiziani

**Affiliations:** 1 Department of Nutritional Sciences, The University of Texas at Austin, Austin, TX USA; 2 Division of Pharmacology and Toxicology, College of Pharmacy, The University of Texas at Austin, Austin, TX USA; 3 Dell Pediatric Research Institute, The University of Texas at Austin, Austin, TX USA; 4 The Brown Foundation Institute of Molecular Medicine, University of Texas Health Science Center at Houston, Houston, TX USA

**Erratum to:** *npj Precision Oncology* (2017); doi:10.1038/s41698-017-0024-z; Published 05 June 2017

The supplemental tables were missing in the original publication. These files have now been added in the HTML of this Article.

