# Supplementary material for: Combinatorial treatment with natural compounds in prostate cancer inhibits prostate tumor growth and leads to key modulations of cancer cell metabolism
Source: NPJ Precis Oncol. 2017 Jun 5;1:18. doi: 10.1038/s41698-017-0024-z (PMC5705091; doi:10.1038/s41698-017-0024-z)
Supplement: Supplementary file 1 — Supplemental Information [file 41698_2017_24_MOESM1_ESM.docx]

**SUPPLEMENTARY INFORMATION**

**Screening of natural compounds for identification of synergistic combinations in prostate cancer**

Alessia Lodi^1,5^, Achinto Saha^2,5^, Xiyuan Lu^1^, Bo Wang^1^, Enrique Sentandreu^1^, Meghan Collins^1^, Mikhail G Kolonin^4^, John DiGiovanni^2,3,*^, Stefano Tiziani^1,3,*^

^1^ Department of Nutritional Sciences, The University of Texas at Austin, Austin, Texas

^2^ Division of Pharmacology and Toxicology, College of Pharmacy, The University of Texas at Austin, Austin, Texas

^3^ Dell Pediatric Research Institute, The University of Texas at Austin, Austin, Texas

^4^ The Brown Foundation Institute of Molecular Medicine, University of Texas Health Science Center at Houston, Houston, Texas

^5^ These authors contributed equally to this work

^*^ Corresponding author

**SUPPLEMENTARY RESULTS**

The intracellular lipid fraction from HMVP2 cell extracts was analyzed using UHPLC-MS. The results were first analyzed by PCA of all lipid features. The scores plot (data not shown) indicated trends very similar to the results observed for the polar compounds. While the detailed analysis of the perturbation of all lipids might facilitate a better understanding of the mechanism of action of the individual and combined treatments, due to the high complexity and to the difficulty to distinguish and identify the exact molecular structure of each lipid metabolite, we performed the analysis by assigning the metabolites to different lipid classes, namely fatty acyls (FA), polyketides (PK), prenol lipids (PR), sterol lipids (ST), sphingolipids (SP), glycerophospholipids (GP) and glycerolipids (GL). In addition, to decrease the likelihood of false discovery, we focused lipids in 12 sub classes of SP, GP and GL (**Supplementary Table 4**) which have high confidence in identification. Notably, and in line with polar metabolite results included above, among the individual treatments UA showed major changes compared to control cells, while changes in lipid metabolites following RES and, even more so, CUR were limited. Nevertheless, the combination of CUR+UA highly upregulated ceramide, cardiolipin, glucosylceramide and phosphatidylinositol, and downregulated phosphatidylglycerol significantly and to a much greater extent than UA alone. In line with the results of the polar metabolite analysis, the combination of UA+RES induced effects similar to CUR+UA, but with overall smaller changes.

**SUPPLEMENTARY MATERIALS and METHODS**

***HMVP2 cell extracts for metabolomics and isotopic labeling analysis***

HMVP2 cells were treated for 12 hours with either solvent control (DMSO) or UA, CUR and RES administered alone or in combination. For the isotopic labeled glutamine flux analysis, unlabeled (natural abundance) glutamine was completely replaced with 13C5,15N2-glutamine (Cambridge Isotopes Laboratories, Inc.) in the medium. At the end of the 12-hours treatment period, an aliquot of media was collected from each flask for extracellular analysis. Cells were washed twice with PBS, harvested and snap-frozen in liquid nitrogen as previously reported^1-4^. The extraction of intracellular metabolites was performed using a modified Bligh-Dyer procedure by adding water, methanol and chloroform in equal volumes (final solution 1∶1∶1 water:methanol:chloroform). The solution was vortexed vigorously to extract the intracellular metabolites and then centrifuged to separate the aqueous and lipid phases. The two phases were then collected separately and dried. Four replicates per treatment-condition were collected for MRS and MS-based metabolomics analyses.

***Mice serum for metabolomics analysis***

Mice whole blood was collected at the time of sacrifice by cardiac puncture and allowed to clot at room temperature. Serum was collected following centrifugation and stored at -80 °C. Prior to the metabolomics analysis the serum was deproteinized by ultrafiltration using 3 kDa Nanosep centrifugal device (Pall Corporation).

***MRS-based metabolomics analysis***

One dimensional (1D) ^1^H MRS spectra were acquired on a Bruker Avance III 500 MHz with 1.7 mm TCI MicroCryoProbe system (Bruker BioSpin Corp., Billerica, MA) equipped with autosampler at 300 K. The water resonance was suppressed using the excitation sculpting pulse sequence^5^ and at least 128 scans and 8 dummy scans, 32768 data points were acquired with a spectral width of 8012.820 Hz. Raw data were processed using NMRLab and MetaboLab^6,7^ in the MATLAB programming environment (MathWorks Inc., Natick, MA). Post processing of spectra included scaling according to the probabilistic quotient method (PQN)^8^, segment alignment^9^, exclusion of selected signals arising from solvents and TMSP, and application of a generalized log transformation^10^. Metabolite assignment and quantification were performed using Chenomx 8 NMR Suite (Chenomx Inc., Edmonton, Alberta, Canada), the Birmingham Metabolite Library^11^, and the Human Metabolome Database^12^.

***Solvents and reagents for MS experiments***

All chemicals were HPLC grade and all the aqueous solutions were prepared using ultrapure water (Milli-Q system, Millipore, Billerica, MA). Formic acid, acetonitrile, isopropanol, ammonium formate, methanol, chloroform, sodium azide, sodium dodecyl sulfate, caffeine, caffeine fragment, monosodium phosphate, and disodium phosphate, ammonium acetate and mass spectrometry calibration mixtures (Pierce LTQ Velos ESI Positive and Negative Ion calibration solutions) were purchased from Thermo Fisher Scientific (Pittsburgh, PA, USA). Compounds for customized calibration, including pyruvic acid, sodium taurocholate, n-butylamine, and Met-Arg-Phe-Ala pentapeptide (MRFA) were purchased from Sigma-Aldrich (St. Louis, MO, USA). Water soluble internal standards, including cysteine (3,3-D2, 98%), fumaric acid (2,3-D2, 98%), DL-glutamic acid (2,4,4-D3, 98%), L-tryptophan (indole-D5, 98%), D4 cystine, and succinic acid (D4, 98%) were bought from Cambridge Isotope Laboratories (Tewksbury, MA, USA). Apolar internal standards for mass spectrometry included 1,2-dipalmitoyl-d62-sn-glycero-3-phosphoethanolamine (16:0 PE-d62), 1,2-diphytanoyl-sn-glycero-3-phosphoethanolamine (4ME 16:0 PE), 1,2-diphytanoyl-sn-glycero-3-phospho-L-serine (4ME 16:0 PS), 1,2-dimyristoyl-d54-sn-glycero-2-[phospho-L-serine] sodium salt (14:0 PS-d54), 1-myristoyl-2-hydroxy-sn-glycero-3-phosphocholine (Lyso PC), 1,2-dipalmitoyl-d62-sn-glycero-3-phosphocholine-1,1,2,2-d4-N,N,N-trimethyl-d9 (16:0 PC-d75), 1,2-dilauroyl-sn-glycero-2-phosphocholine (12:0 PC), 1,2-dioleoyl-sn-glycero-3-phosphoinositol ammonium salt (18:1 PI), 1,2-dilauroyl-sn-glycerol (12:0 DG), and Ceramide/Sphingoid Internal Standard Mixture I and were all acquired from Avanti Polar Lipids Inc. (Alabaster, Alabama). In addition, heptadecanoic-17,17,17-d3 acid was purchased from CDN Isotopes (Pointe-Claire, Quebec, Canada). Deuterium oxide (D_2_O, 99.8%) for NMR data acquisition was purchased from Acros Organics (Fair Lawn, NJ). Deuterated 3-(trimethylsilyl)-2,2,3,3-tetradeuteropropionic acid (TSP) as an NMR internal standard (IS) was purchased from Cambridge Isotope Laboratories (Tewksbury, MA).

***MS-based metabolomics analysis***

Chromatographic separation was performed on a Thermo Scientific (Thermo Fisher Sci., San José, CA, USA) Accela UHPLC system equipped with a quaternary pump, vacuum degasser and an open autosampler with a temperature controller. For the polar phase and media, the column loaded was a 100 mm x 2.1 mm, 5 µm, ZIC-pHILIC polymeric column (Millipore Co., Darmstadt, Germany). Separation conditions were: injection volume, 3 µL; flow rate, 0.3 mL/min; autosampler temperature, 6 °C; oven temperature, 21 °C; solvent A, water/formic acid (99.9:0.1, v/v); solvent B, acetonitrile/formic acid (99.9:0.1, v/v); separation gradient, initially 96% B, linear 96-20% B in 15 min, purging with 1% B for 5 min and column equilibration with 96% B for 10 minutes. Mass spectrometry analysis was carried out on a Q Exactive benchtop Orbitrap detector loading an electrospray (ESI) source simultaneously operating in fast negative/positive polarity switching ionization mode. The detector run in Full scan MS analysis under the following conditions: spray voltage, 4.0 kV; capillary temperature, 300℃; sheath gas, 55 (arbitrary units); auxiliary gas, 30 (arbitrary units); microscans, 1; AGC target, 1e^6^; maximum injection time, 100 ms; mass resolution, 70,000; considered *m/z* range, 115-450.

For the apolar phase, lipids were separated on a Kinetex 2.6 μm C-18 100 Å column (Phenomenex, Torrance, CA). An 80:20 solution of water and acetonitrile with 10 mM ammonium acetate and 0.05% (w/v) formic acid was used for mobile phase A. For mobile phase B, a solution of 90:9:1 isopropanol:acetonitrile:water with 10 mM ammonium acetate and 0.5% (w/v) formic acid was prepared. The gradient was set with a flow rate of 260 ul/min in a total of 30 min. HESI probe has spray voltage of 3.5 kV, capillary temperature, 250°C，sheath gas, 25 (arbitrary units); auxiliary gas, 15 units (arbitrary units).

To ensure the accuracy of the MS analysis, the detector was calibrated through the commercial calibration solutions provided by the manufacturer followed by a customized adjustment for small molecular masses. Control of the UHPLC-MS system and data acquisition were managed by the Xcalibur (ThermoScientific, San Jose, CA, USA) v. 2.2 software.

A pooled sample, representing the combined composition of all samples, was prepared for quality control (QC) to control for possible instrumental error (drift) in data acquisition^13,14^. The QC samples were ran once every five samples.

All raw MS datasets were processed using Sieve 2.2 (Thermo Fisher Scientific). Only the positive and negative ionization-mode features with coefficient of variation (CV) lower than 25% in the QC samples were considered for further analysis. Peaks were scaled according to PQN and internal standards levels. Featured were then mined against an in house database of accurate masses and retention times generated in our laboratory using the IROA 300, Mass Spectrometry Metabolite Library of Standards (MSMLS; IROA Technologies, Bolton, MA). In addition, databases of accurate masses taken from the Kyoto Encyclopedia of Genes and Genomes (KEGG)^15^ and the Human Metabolome database^12^ were also mined. MS data were then combined to the MRS data for the subsequent post-processing, followed by univariate and multivariate statistical analyses.

**Western blotting protein detection**

HMVP2 cells were treated with the indicated concentrations of CUR, UA, RES or their combinations for the specified time (2, 6, and 24 h). After incubation, cells were washed with ice-cold PBS and lysed in Radioimmunoprecipitation assay (RIPA) buffer. Proteins were separated by 4-15% SDS-PAGE gel and transferred to a nitrocellulose membrane. After blocking in 5% bovine serum albumin (BSA) for 1 hour, the membranes were probed with specific primary antibodies (listed below) overnight at 4°C. Following secondary antibody incubation, the membranes were visualized using a chemiluminescent detection kit (Pierce Biotechnology). Western blots were quantified by densitometry analysis using the freeware ImageJ (National Institutes of Health, Bethesda, MD).

The following antibodies were used for western blotting: STAT3 (Cell signaling 9139), pSTAT3Ser727 (Cell signaling 9134), pSTAT3Tyr705 (Cell signaling 9145), Src (Cell signaling 2109), pSrcTyr416 (Cell signaling 2101), AMPK (Cell signaling 2532), pAMPKThr172 (Cell signaling 2531), p70S6K (Cell signaling 9202), pp70S6KThr389 (Cell signaling 9234), S6 Ribo (Cell signaling 2217), pS6 RiboS235/236 (Cell signaling 2211), pS6 RiboS240/244 (Cell signaling 5364), GAPDH (Cell signaling 5174), PARP (Cell signaling 9542), Caspase 9 (Cell signaling 9508), Cleaved Caspase 9 (Cell signaling 7237), ASCT2 (Cell signaling 8057) and β-actin (Sigma-Aldrich A5316). Secondary antibodies were purchased from GE Healthcare.

**Supplementary References**

1 Guma, M. *et al.* Targeting Glycolysis in Rheumatoid Arthritis. *Arthritis & Rheumatology* **66**, S416-S416 (2014).

2 Lodi, A. *et al.* Hypoxia Triggers Major Metabolic Changes in AML Cells without Altering Indomethacin-Induced TCA Cycle Deregulation. *Acs Chemical Biology* **6**, 169-175, doi:10.1021/cb900300j (2011).

3 Tiziani, S. *et al.* Metabolomic Profiling of Drug Responses in Acute Myeloid Leukaemia Cell Lines. *PloS one* **4**, doi:10.1371/journal.pone.0004251 (2009).

4 Lodi, A. & Ronen, S. M. Magnetic resonance spectroscopy detectable metabolomic fingerprint of response to antineoplastic treatment. *PloS one* **6**, e26155 (2011).

5 Hwang, T. L. & Shaka, A. J. Water Suppression That Works - Excitation Sculpting Using Arbitrary Wave-Forms and Pulsed-Field Gradients. *J Magn Reson Ser A* **112**, 275-279, doi:DOI 10.1006/jmra.1995.1047 (1995).

6 Gunther, U. L., Ludwig, C. & Ruterjans, H. NMRLAB-Advanced NMR data processing in matlab. *J Magn Reson* **145**, 201-208, doi:10.1006/jmre.2000.2071 (2000).

7 Ludwig, C. & Günther, U. L. MetaboLab - advaced NMR data processing and analysis for metabolomics. *Bmc Bioinformatics* **12** (2011).

8 Dieterle, F., Ross, A., Schlotterbeck, G. & Senn, H. Probabilistic quotient normalization as robust method to account for dilution of complex biological mixtures. Application in 1H NMR metabonomics. *Anal Chem* **78**, 4281-4290, doi:10.1021/ac051632c (2006).

9 Savorani, F., Tomasi, G. & Engelsen, S. B. icoshift: A versatile tool for the rapid alignment of 1D NMR spectra. *Journal of Magnetic Resonance* **202**, 190-202 (2010).

10 Parsons, H. M., Ludwig, C., Gunther, U. L. & Viant, M. R. Improved classification accuracy in 1- and 2-dimensional NMR metabolomics data using the variance stabilising generalised logarithm transformation. *BMC Bioinformatics* **8**, 234, doi:10.1186/1471-2105-8-234 (2007).

11 Ludwig C, E. J., Lodi A, Tiziani S, Manzoor SE, et al. Birmingham Metabolite Library: a publicly accessible database of 1-D H-1 and 2-D H-1 J-resolved NMR spectra of authentic metabolite standards (BML-NMR). *Metabolomics* **8**, 8-18 (2012).

12 Wishart, D. S. *et al.* HMDB: the Human Metabolome Database. *Nucleic Acids Res* **35**, D521-526, doi:10.1093/nar/gkl923 (2007).

13 Sangster, T., Major, H., Plumb, R., Wilson, A. J. & Wilson, I. D. A pragmatic and readily implemented quality control strategy for HPLC-MS and GC-MS-based metabonomic analysis. *Analyst* **131**, 1075-1078, doi:10.1039/b604498k (2006).

14 Want, E. J. *et al.* Global metabolic profiling of animal and human tissues via UPLC-MS. *Nature Protocols* **8**, 17-32, doi:10.1038/nprot.2012.135 (2013).

15 Okuda, S. *et al.* KEGG Atlas mapping for global analysis of metabolic pathways. *Nucleic Acids Res* **36**, W423-W426, doi:10.1093/nar/gkn282 (2008).
