# Supplementary material for: Combinatorial treatment with natural compounds in prostate cancer inhibits prostate tumor growth and leads to key modulations of cancer cell metabolism
Source: NPJ Precis Oncol. 2017 Jun 5;1:18. doi: 10.1038/s41698-017-0024-z (PMC5705091; doi:10.1038/s41698-017-0024-z)
Supplement: Supplementary file 2 — Supplemental Figure Legend [file 41698_2017_24_MOESM2_ESM.docx]

**[Supplemental Figure Legend](https://static-content.springer.com/esm/art%3A10.1038%2Fs41698-017-0011-4/MediaObjects/41698_2017_11_MOESM1_ESM.docx)**

**Screening of natural compounds for identification of synergistic combinations in prostate cancer**

Alessia Lodi^1,5^, Achinto Saha^2,5^, Xiyuan Lu^1^, Bo Wang^1^, Enrique Sentandreu^1^, Meghan Collins^1^, Mikhail G Kolonin^4^, John DiGiovanni^2,3,*^, Stefano Tiziani^1,3,*^

**Supplementary Figure 1: Comparison of the effect of therapeutic agents or combination of UA with CUR or RES on the growth/survival of PCa cells**. Isolated mouse (HMVP2) and human (LNCaP and PC-3) PCa cells (1–5 × 10^3^/well) were plated in 96 well tissue culture plates and treated with the indicated concentration of abiraterone, enzalutamide, docetaxel, rapamycin, UA+RES or UA+CUR for 48h and cell growth/survival was measured by MTT assay. One-way ANOVA with significance at p<0.05 was used. *P<0.05, **P<0.01, ***P<0.001, ****P<0.0001.

**Supplementary Figure 2. *In vivo* effect of docetaxel on HMVP2 allograft tumor and *in vitro* cell growth.** HMVP2 cell spheroids were injected subcutaneously into the flank of male FVB/N mice. 4 days after spheroid injection mice were treated (half of the drug volume intraperitoneally and the other half subcutaneously around the tumor) weekly with docetaxel (20 mg/kg body weight) for 4 consecutive weeks. Tumor volume (A) and tumor weight (B) are shown as mean ± SEM. Cell viability (as ATP bioluminescence, C) was measured *in vitro* in NMVP and HMVP2 cells treated with a range of doses (1 nM to 250 nM) of docetaxel for 12 hours. ATP bioluminescence values were normalized to control and shown as mean ± SEM. . Two tailed Student’s t-test was performed; all comparisons were not significantly different (p>0.05).

**Supplementary Figure 3. Effect of treatment on relevant signaling pathways.** HMVP2 cells were treated for 6 or 24 hours with 20 μM CUR, UA, RES or their combinations of 2 compounds. Phospho and total protein levels for STAT3, p70S6K, S6 and AMPK were measured by Western blotting. Western blotting were performed two times with β-actin controls for each experiment. Numbers above blots indicate band intensities (normalized to control).

**Supplementary Figure 4. Effect of treatment with metformin and rapamycin on AMPK and mTORC1 signaling pathways.** HMVP2 and LNCaP cells were treated for 24 hours with 0.5 mM metformin or 1 nM rapamycin. The levels of the phospho- and total protein levels for AMPK, p70S6K and S6 were measured by Western blotting. Numbers above blots indicate band intensities (normalized to control).

**Supplementary Figure 5. The combination of CUR, UA and RES induces Stat3 inhibition and apoptosis in DU145 cells.** DU145 cells were treated for 24 hours with the indicated concentrations of CUR, UA, RES or their combinations of 2 compounds. The levels of the phospho- and total protein levels for STAT3, and of the apoptosis markers were measured by Western blotting (A). The percent of apoptotic cells (B) were measured by Annexin V staining. One-way ANOVA with significance at p<0.05 was used. Statistical significance is shown as different from control (a), CUR (b), UA (c) and RES (d)
