# Supplementary figures and images for: Combinatorial treatment with natural compounds in prostate cancer inhibits prostate tumor growth and leads to key modulations of cancer cell metabolism
Source: NPJ Precis Oncol. 2017 Jun 5;1:18. doi: 10.1038/s41698-017-0024-z (PMC5705091; doi:10.1038/s41698-017-0024-z)

Supplementary Figure 1

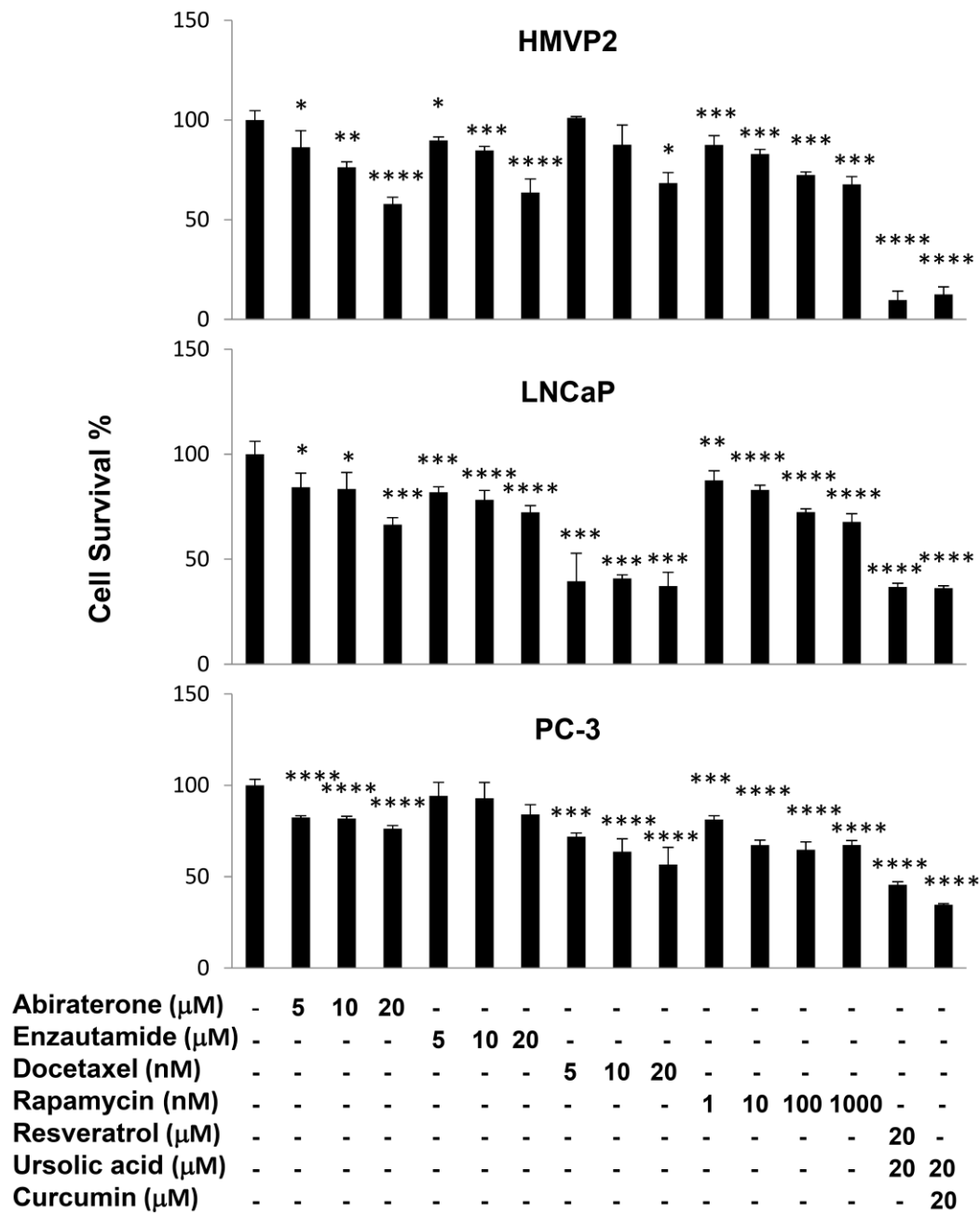

Supplement: Supplementary file 3 — Supplementary Figure 1 [file 41698_2017_24_MOESM3_ESM.pdf]

Supplementary Figure 2

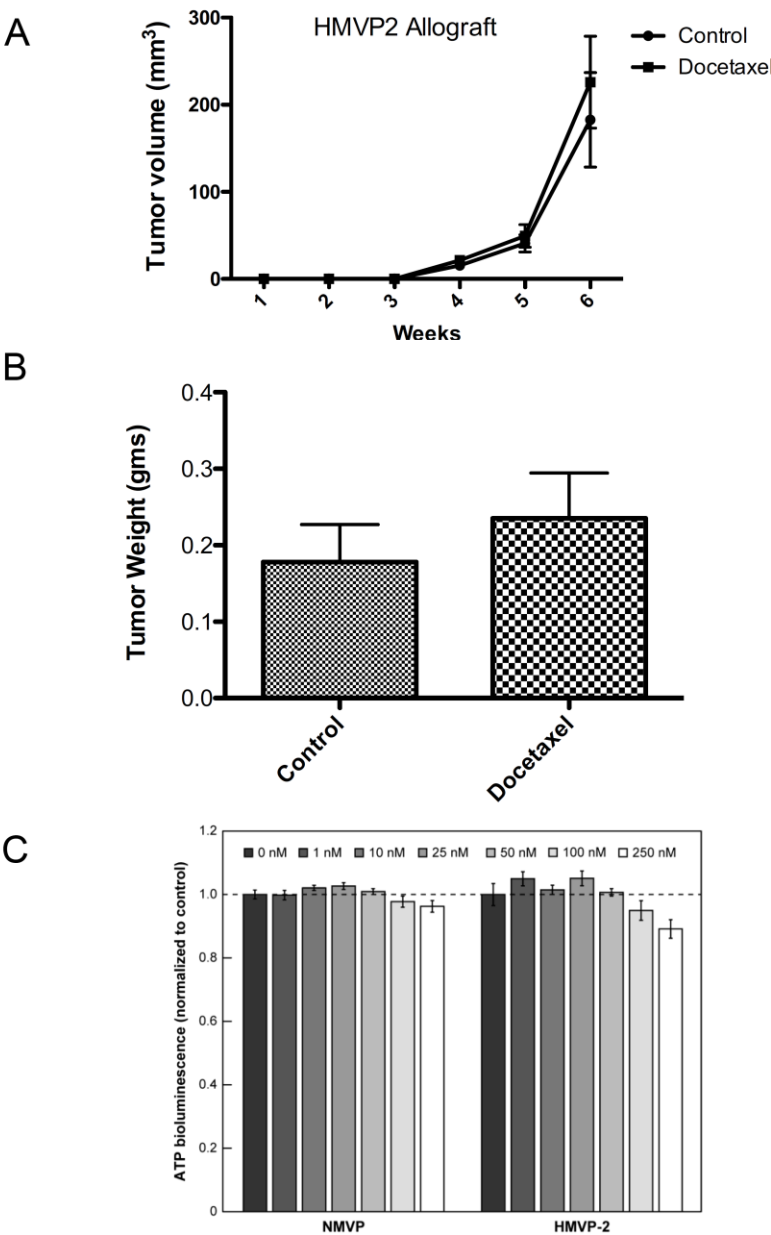

Supplement: Supplementary file 4 — Supplementary Figure 2 [file 41698_2017_24_MOESM4_ESM.pdf]

Supplementary Figure 3

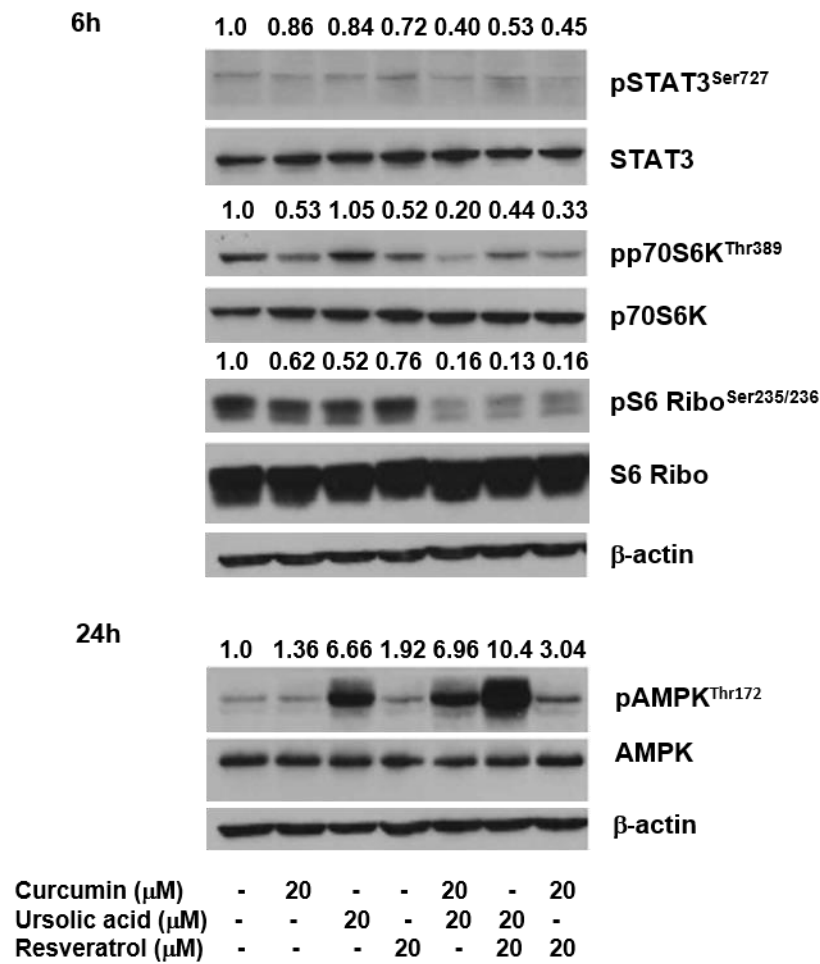

Supplement: Supplementary file 5 — Supplementary Figure 3 [file 41698_2017_24_MOESM5_ESM.pdf]

Supplementary Figure 4

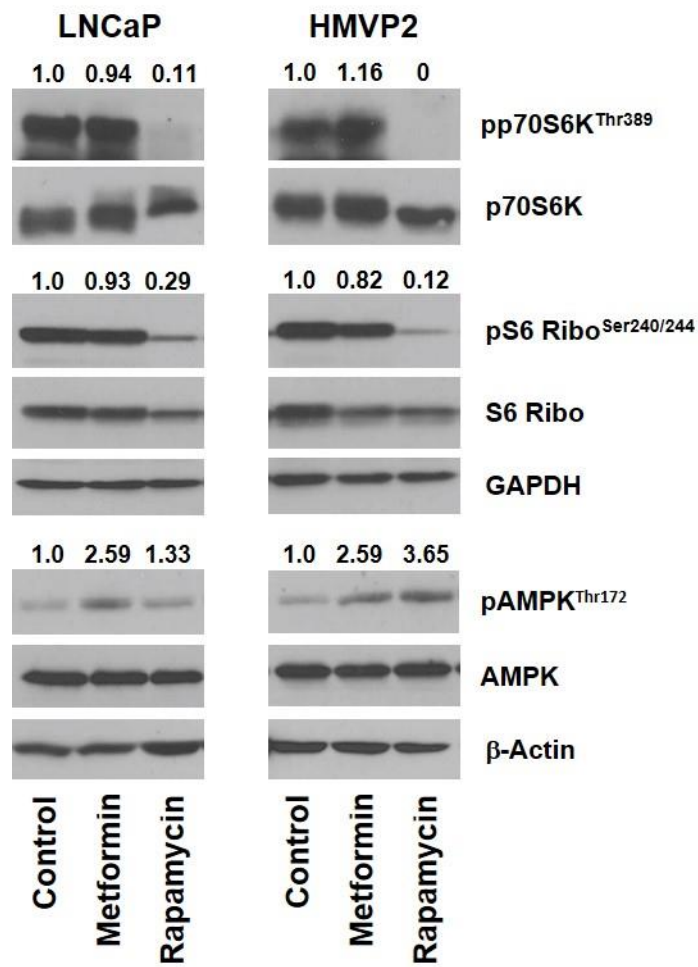

Supplement: Supplementary file 6 — Supplementary Figure 4 [file 41698_2017_24_MOESM6_ESM.pdf]

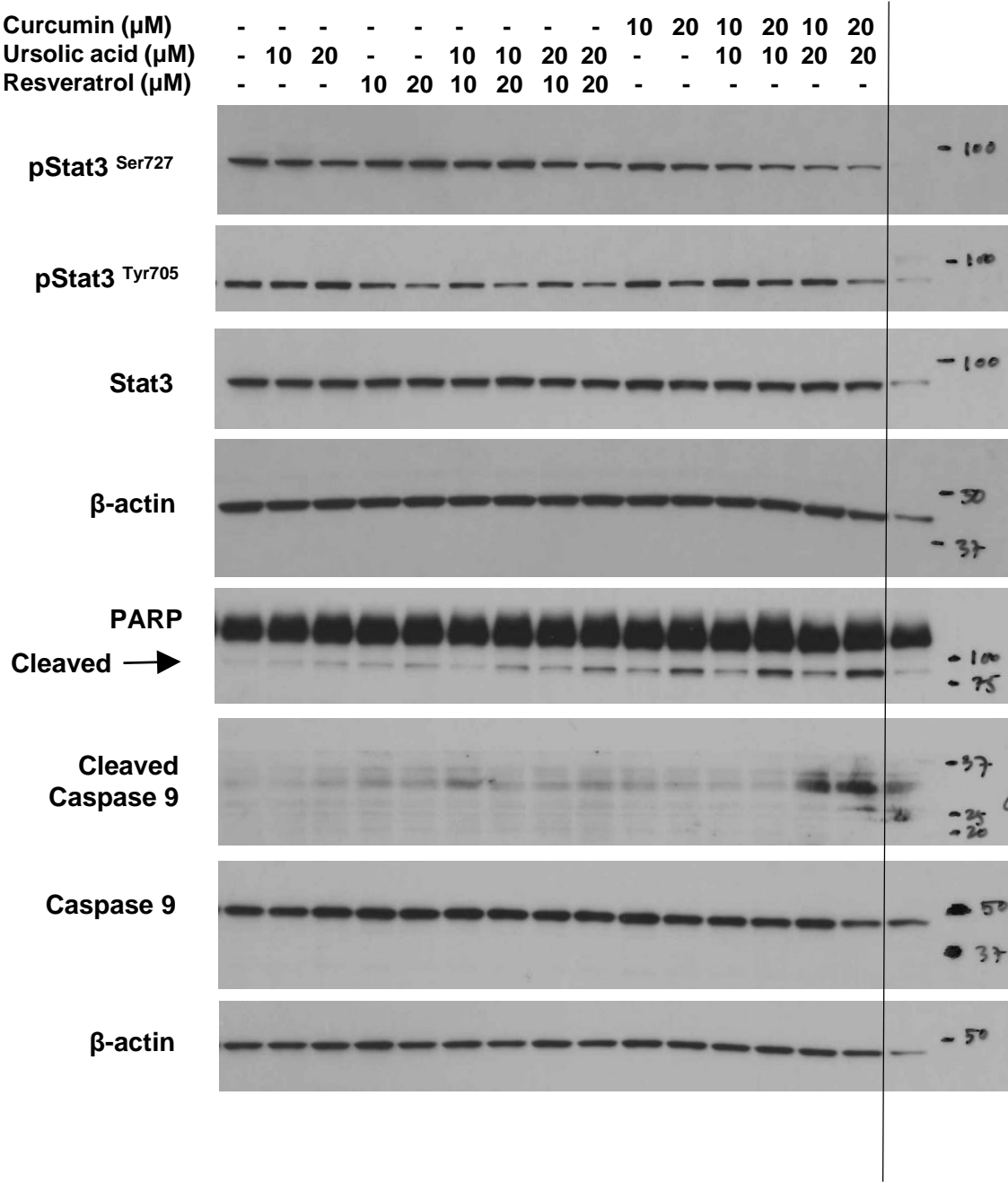

Supplement: Supplementary file 7 — Supplementary Figure 5 [file 41698_2017_24_MOESM7_ESM.pdf]
