# Supplementary material for: Combinatorial treatment with natural compounds in prostate cancer inhibits prostate tumor growth and leads to key modulations of cancer cell metabolism
Source: NPJ Precis Oncol. 2017 Jun 5;1:18. doi: 10.1038/s41698-017-0024-z (PMC5705091; doi:10.1038/s41698-017-0024-z)
Supplement: Supplementary file 8 — Supplementary Tables [file 41698_2017_24_MOESM8_ESM.pdf]

Supplementary Table 1. ATP bioluminescence values (normalized to control) and z-factor derived from the ATP values in HMVP2 cells treated with the individual natural compounds listed at the doses of 5, 10 or 20 µM and for 12, 24 or 48 hours. Column 1 on the left indicates the order of the natural compounds used to sort ATP levels in Figure 1. Natural compounds are listed in alphabetical order.

		5 µM treatment dose						10 µM treatment dose						20 µM treatment dose					
		12 hours		24 hours		48 hours		12 hours		24 hours		48 hours		12 hours		24 hours		48 hours	
		ATP	z-factor	ATP	z-factor	ATP	z-factor	ATP	z-factor	ATP	z-factor	ATP	z-factor	ATP	z-factor	ATP	z-factor	ATP	z-factor
80	(-)-Epigallocatechin gallate	0.69	-1.68	0.69	-1.99	0.66	-1.22	0.44	-0.14	0.46	-1.28	0.49	-1.58	0.61	-1.16	0.59	-1.24	0.74	-2.66
19	(+)-Usniacin (D-Usnic acid)	0.69	-1.79	0.67	-2.02	0.71	-1.76	0.47	-0.81	0.62	-3.02	0.52	-1.74	0.27	0.39	0.45	-0.04	0.49	-0.80
24	10-Hydroxycamptothecin	0.96	-12.57	0.42	0.41	0.11	0.77	0.84	-2.27	0.36	-0.08	0.24	0.69	0.65	0.24	0.43	0.36	0.48	0.54
38	3-Indolebutyric acid (IBA)	0.73	-2.32	0.73	-2.97	0.71	-2.38	0.51	-0.22	0.52	-1.68	0.51	-1.53	0.53	-0.13	0.65	-1.61	0.96	-28.47
86	4-Methylumbelliferone (4-MU)	0.78	-3.46	0.71	-1.81	0.74	-2.98	0.68	-2.34	0.64	-2.08	0.60	-1.95	0.65	-1.24	0.72	-1.84	0.92	-21.59
35	5-hydroxytryptophan (5-HTP)	0.90	-3.19	0.96	-10.52	1.01	-26.01	0.89	-1.87	1.01	-8.00	0.97	-10.82	0.73	-0.10	0.87	-1.94	0.97	-10.30
49	Aesculin (Esculin)	0.78	-2.75	0.77	-3.13	0.72	-2.42	0.52	-0.28	0.57	-1.74	0.59	-2.59	0.53	-0.45	0.66	-1.66	0.90	-8.83
36	Aloin (Barbaloin)	0.95	-20.80	1.02	-91.05	0.97	-4.73	1.01	-64.72	1.12	-54.86	1.08	-8.24	0.61	-0.12	0.93	-2.25	1.17	-2.20
99	Ammonium Glycyrrhizinate (AMGZ)	0.95	-15.11	0.90	-5.05	0.90	-1.37	0.93	-5.06	0.95	-6.10	0.82	-1.34	0.71	-1.64	0.80	-0.46	0.86	-1.95
11	Amygdalin	0.26	0.07	0.20	0.46	0.13	0.54	0.09	0.66	0.17	-0.07	0.08	0.76	0.06	0.71	0.12	0.52	0.12	0.39
30	Andrographolide	0.80	-3.84	0.48	0.17	0.69	-1.68	0.49	-0.14	0.37	-0.87	0.35	-0.79	0.42	0.01	0.34	-0.16	0.38	-0.84
81	Apigenin	0.81	-3.43	0.77	-2.47	0.78	-1.77	0.72	-2.98	0.64	-2.51	0.53	-1.14	0.60	-1.18	0.79	-3.41	0.92	-11.39
79	Apocynin (Acetovanillone)	1.08	-2.72	0.99	-18.96	0.87	-2.22	1.02	-23.46	1.02	-13.61	0.83	-2.44	0.82	-1.15	1.04	-11.45	1.23	-0.62
53	Arbutin (Uva, p-Arbutin)	0.88	-9.12	0.80	-3.65	0.69	-1.02	0.57	-0.89	0.60	-2.55	0.56	-1.98	0.60	-0.55	0.67	-1.86	0.90	-11.61
75	Artesunate	0.86	-7.27	0.59	-0.90	0.30	0.39	0.74	-3.36	0.48	-1.16	0.24	0.56	0.62	-1.06	0.52	-0.29	0.61	-0.79
14	Asiatic acid	0.90	-11.77	0.76	-3.77	0.68	-1.25	0.37	-0.09	0.38	-0.83	0.48	-2.06	0.16	0.57	0.40	-0.11	0.52	-2.08
34	Astragaloside A	1.10	-3.58	0.93	-3.02	0.91	-3.10	0.93	-4.71	1.07	-42.57	0.98	-34.90	0.72	-0.09	1.09	-3.06	1.13	-1.87
68	Azomycin (2-Nitroimidazole)	0.84	-6.46	0.78	-4.01	0.77	-4.15	0.68	-2.42	0.58	-2.05	0.38	-0.48	0.66	-0.92	0.66	-1.28	0.83	-10.15
56	Baicalein	0.85	-5.70	0.72	-3.26	0.67	-2.01	0.49	-0.32	0.47	-1.69	0.36	-0.64	0.62	-0.66	0.62	-1.94	0.62	-1.63
109	Baicalin	0.85	-6.70	0.82	-4.98	0.78	-3.48	0.73	-3.05	0.62	-2.47	0.51	-2.25	0.75	-2.17	0.75	-1.87	0.84	-8.31
103	Berberine Hydrochloride	0.83	-6.42	0.80	-5.14	0.72	-2.19	0.73	-2.17	0.77	-5.00	0.74	-5.49	0.66	-1.85	0.95	-21.39	1.32	-7.16
65	Bergenin (Cuscutin)	0.88	-10.00	0.79	-5.11	0.71	-2.07	0.56	-0.80	0.58	-2.75	0.56	-3.04	0.63	-0.87	0.69	-2.61	0.66	-2.79
37	Betulinic acid	0.80	-0.44	0.88	-1.57	0.85	-2.04	0.50	0.46	0.61	-0.45	0.66	-0.76	0.75	-0.13	0.96	-14.13	0.96	-7.46

124	Bilobalide	0.84	-6.46	0.90	-9.03	0.81	-3.04	0.74	-2.28	0.66	-3.00	0.46	-1.09	0.80	-4.27	0.74	-2.03	0.81	-8.18
25	Biochanin A (4-Methylgenistein)	1.03	-13.38	0.89	-3.78	0.76	-0.40	0.99	-108.97	0.49	-0.96	0.21	0.78	0.50	0.19	0.43	0.39	0.34	0.56
40	Butylscopolamine bromide (Scopolamine butylbromide)	1.02	-13.32	0.45	0.17	0.15	0.75	0.86	-1.62	0.32	0.06	0.18	0.84	0.67	-0.35	0.29	0.57	0.30	0.61
77	Caffeic acid	0.87	-7.73	0.81	-5.11	0.72	-2.45	0.59	-0.82	0.56	-2.66	0.50	-2.03	0.62	-1.12	0.68	-2.43	0.89	-12.54
15	Caffeic acid phenylethyl ester	0.74	-0.66	0.53	0.30	0.52	0.21	0.64	-0.41	0.31	0.11	0.32	0.75	0.34	0.56	0.36	0.60	0.37	0.26
117	Chlorogenic acid	0.85	-6.52	0.88	-7.77	0.86	-7.27	0.78	-2.87	0.66	-3.13	0.45	-1.68	0.83	-3.39	0.92	-46.64	0.87	-9.47
101	Chrysin	0.85	-6.35	0.88	-6.78	0.79	-2.48	0.65	-1.14	0.70	-3.99	0.54	-2.53	0.65	-1.78	0.82	-5.25	0.93	-11.95
42	Chrysophanic acid (Chrysophanol)	1.04	-9.43	0.30	0.50	0.10	0.73	0.79	-0.85	0.30	0.07	0.16	0.75	0.70	-0.37	0.36	0.57	0.28	0.70
102	Cinchonidine	1.06	-7.82	0.99	-26.41	1.03	-11.74	0.71	-2.89	0.97	-7.01	0.82	-2.98	0.79	-1.82	0.87	-1.42	0.92	-2.94
6	Corosolic acid (CA)	1.03	-9.80	0.89	-3.47	1.05	-10.03	0.41	0.46	0.50	-0.82	0.63	-0.67	0.12	0.76	0.40	0.58	0.36	0.54
27	Cryptotanshinone	0.77	-0.37	0.65	0.21	0.87	-0.91	0.65	-4.73	0.37	-0.39	0.36	0.32	0.43	0.09	0.41	0.29	0.44	0.29
5	Curcumin	0.87	-1.79	0.67	-0.46	0.87	-1.32	0.34	0.60	0.49	-0.41	0.52	0.32	0.09	0.76	0.13	0.72	0.49	0.44
32	Curcumol	1.14	-1.47	0.84	-2.08	0.54	-0.73	0.89	-1.21	0.36	-0.06	0.18	0.75	0.66	-0.06	0.34	0.58	0.31	0.62
93	Cyclocytidine HCl	0.76	-2.01	0.36	-0.23	0.16	0.80	0.63	-2.57	0.17	0.09	0.09	0.79	0.72	-1.44	0.17	0.69	0.11	0.74
57	Cyclosporin A (Cyclosporine A)	0.96	-24.79	0.76	-0.54	0.58	0.40	0.52	-0.42	0.74	-1.62	0.42	0.29	0.70	-0.69	0.68	-0.50	0.62	0.38
140	Cytisine (Baphitoxine, Sophorine)	0.89	-3.79	0.88	-1.62	0.94	-4.40	0.96	-33.66	0.85	-3.38	0.83	-3.81	0.97	-24.99	0.92	-3.82	1.09	-7.86
58	Dihydroartemisinin (DHA)	1.06	-7.99	0.58	0.30	0.28	0.82	0.68	-1.78	0.63	-0.61	0.36	0.71	0.69	-0.69	0.51	0.28	0.60	0.27
95	Dihydromyricetin (Ampeloptin)	0.94	-10.61	0.91	-1.92	0.98	-9.81	0.87	-2.84	1.07	-41.62	0.99	-60.41	0.77	-1.45	0.91	-2.93	1.12	-2.86
2	Dioscin (Collettiside III)	0.11	0.59	0.14	0.71	0.09	0.83	0.06	0.83	0.13	0.24	0.10	0.85	0.03	0.81	0.04	0.79	0.05	0.81
50	Diosmetin (Luteolin 4-methyl ether)	1.11	-3.76	0.97	-14.16	0.90	-1.76	1.05	-12.76	0.82	-2.27	0.70	-0.12	0.59	-0.46	0.58	0.29	0.40	0.51
121	Diosmin	0.96	-10.62	0.91	-1.69	0.95	-7.57	0.94	-26.55	0.85	-3.05	0.88	-5.25	0.83	-3.82	0.63	-0.02	0.96	-6.19
71	DL-Carnitine hydrochloride	1.06	-6.71	0.88	-2.14	0.89	-1.37	0.67	-2.12	0.86	-2.32	0.80	-2.86	0.77	-0.99	0.83	-0.72	0.87	-2.95
92	D-Mannitol (Osmitrol)	1.04	-9.60	1.18	-1.88	1.07	-2.98	1.21	-3.84	1.40	-5.22	1.03	-21.76	0.76	-1.43	0.88	-1.76	1.07	-5.65
46	Emodin	0.90	-4.74	0.90	-1.81	0.87	-0.92	0.72	-5.41	0.70	-1.25	0.85	-6.46	0.65	-0.41	0.71	0.11	1.17	-1.93
21	Enoxolone (Glycyrrhetin)	1.05	-7.55	0.81	-0.47	0.68	-1.09	0.53	-0.49	0.38	-0.04	0.20	0.73	0.34	0.37	0.40	0.66	0.26	0.54
82	Epi-Corosolic acid (ECA)	0.97	-22.08	1.07	-6.52	1.00	-990.96	0.89	-3.13	1.17	-14.14	1.33	-0.46	0.70	-1.18	1.00	-316.60	1.59	0.26
55	Epi-Maslinic acid (EMA)	0.95	-6.13	1.00	-45.02	1.00	-77.25	0.86	-4.18	1.10	-90.77	1.05	-8.34	0.61	-0.66	0.91	-5.26	1.51	0.08
78	Epi-Ursolic acid (EUA)	0.96	-11.95	0.87	-1.72	0.95	-6.51	0.70	-0.46	1.14	-18.84	1.24	-0.91	0.62	-1.13	0.95	-19.13	1.36	-1.59

90	Fisetin (Fustel)	0.98	-224.82	0.88	-2.64	0.85	-0.93	0.88	-11.79	0.73	-1.82	0.66	-0.32	0.78	-1.35	0.65	-0.05	0.77	-0.44
64	Formononetin (Formononetol)	1.06	-7.87	0.96	-8.46	0.93	-5.24	0.71	-2.04	0.83	-2.35	0.89	-4.16	0.64	-0.84	0.86	-1.98	1.08	-3.25
139	Fumalic acid (Ferulic acid)	1.02	-16.13	1.02	-46.08	1.01	-49.82	0.98	-55.31	1.11	-89.98	0.78	-3.86	0.97	-19.79	0.86	-1.69	1.20	-1.68
113	Gastrodin (Gastrodine)	1.05	-9.09	1.01	-125.38	0.97	-10.62	1.04	-14.09	0.96	-7.18	0.97	-24.03	0.77	-2.66	0.79	-0.53	0.88	-2.32
100	Genistein	0.92	-8.06	0.83	-1.62	0.77	-1.85	0.76	0.20	1.10	-437.12	0.70	-0.30	0.78	-1.75	0.84	-3.87	0.59	-0.12
87	Glycyrrhizic acid	1.06	-6.01	1.06	-10.37	0.92	-2.69	0.71	-3.00	0.85	-1.74	0.74	-0.56	0.60	-1.25	0.84	-1.05	0.88	-3.32
12	Gossypol	0.84	-3.01	0.62	-0.01	0.38	0.26	0.57	-4.22	0.28	0.06	0.17	0.71	0.11	0.70	0.28	0.43	0.25	0.54
26	Gramine	1.00	-30.43	0.98	-13.20	0.94	-3.50	0.73	-2.70	0.92	-3.42	0.75	-0.81	0.65	0.11	0.84	-1.89	0.75	-1.35
16	Guanosine	1.06	-4.56	0.96	-9.22	0.85	-2.24	0.98	-21.86	1.05	-21.80	0.84	-3.07	0.54	0.48	1.24	-1.64	1.29	-0.37
29	Gynostemma Extract	0.99	-81.67	0.93	-6.61	0.95	-3.58	0.84	-8.92	0.80	-2.20	0.88	-6.13	0.63	0.02	0.89	-3.96	1.32	-0.93
31	Hematoxylin (Hydroxybrazilin)	0.97	-37.02	1.08	-5.96	0.94	-3.09	0.93	-10.36	1.12	-63.94	0.78	-0.82	0.59	-0.01	0.71	-0.49	0.79	-0.62
73	Hesperetin	1.06	-7.17	1.02	-54.60	0.94	-3.06	0.84	-5.06	0.87	-2.54	0.79	-1.31	0.68	-1.03	0.91	-2.59	1.07	-5.50
131	Hesperidin	1.03	-8.50	1.04	-17.49	1.05	-7.19	0.97	-36.97	0.92	-3.54	0.90	-7.17	1.11	-7.94	0.90	-4.19	1.05	-31.24
7	Honokiol	0.90	-7.71	0.61	0.13	0.76	-0.11	0.20	0.77	0.44	-0.20	0.34	0.16	0.12	0.76	0.30	0.56	0.37	0.21
137	Hordenine	1.07	-5.68	0.99	-15.10	1.02	-16.19	1.12	-2.83	1.02	-10.50	0.96	-6.75	0.95	-16.27	0.86	-0.84	0.84	-1.21
134	Hyodeoxycholic acid (HDCA)	1.02	-13.23	1.09	-3.76	0.94	-1.86	0.92	-15.24	0.92	-4.49	0.87	-4.66	1.09	-10.14	0.91	-4.75	1.29	-1.59
116	Hypoxanthine	1.14	-1.21	1.10	-3.84	1.02	-12.89	0.99	-31.42	1.30	-5.32	0.82	-1.96	0.92	-3.36	1.05	-6.95	1.23	-1.45
61	Icariin	1.00	-25.86	1.06	-7.97	0.88	-1.28	0.87	-6.38	0.88	-2.75	0.92	-6.72	0.72	-0.76	0.87	-2.15	1.03	-10.25
20	Indirubin	0.83	-2.62	0.86	-1.29	0.73	0.25	0.71	-1.84	0.84	-4.96	0.61	-0.34	0.51	0.37	0.64	0.25	0.77	-0.14
129	Indole-3-carbinol	1.00	-23.81	1.11	-4.42	1.06	-2.67	0.93	-17.35	0.94	-4.00	0.91	-6.34	1.07	-5.93	0.95	-8.00	1.25	-1.02
130	Inosine	1.16	-0.88	1.14	-2.20	1.02	-11.34	0.99	-31.78	1.33	-5.38	0.76	-1.27	0.87	-6.62	1.06	-11.74	1.12	-3.85
23	Ipriflavone	1.05	-3.41	1.15	-2.19	1.01	-28.63	0.87	-1.67	1.22	-9.11	1.21	-2.46	0.48	0.28	0.68	-0.12	1.07	-8.42
43	Isoliquiritigenin	0.99	-292.45	0.84	-0.97	0.94	-4.03	0.71	0.27	0.94	-4.83	0.97	-19.32	0.61	-0.38	1.03	-15.41	1.49	-0.01
98	Kaempferol	0.93	-7.32	0.97	-8.90	1.07	-3.08	0.88	-0.73	1.19	-16.81	0.96	-9.93	0.71	-1.62	0.90	-3.26	0.85	-1.09
74	Kinetin (6-Furfuryladenine)	0.89	-3.72	0.92	-3.72	0.91	-3.67	0.75	-0.29	0.86	-2.20	0.90	-2.83	0.83	-1.03	1.13	-1.61	1.17	-1.51
97	L (+)-Rhamnose Monohydrate	0.86	-1.83	0.92	-3.32	0.99	-22.51	0.73	-0.51	1.02	-13.59	0.85	-0.86	0.69	-1.60	0.81	-0.76	0.85	-2.58
132	Lappaconite Hydrobromide	1.08	-5.17	1.02	-54.11	0.96	-5.16	1.16	-2.66	0.99	-9.05	0.86	-1.74	0.90	-8.42	0.79	-0.16	0.68	-0.50
45	Limonin	0.86	-3.60	0.92	-3.48	0.95	-6.23	0.75	-0.55	0.83	-1.51	0.90	-2.26	0.73	-0.40	0.96	-13.84	1.28	-1.62
54	Luteolin	0.88	-2.03	0.90	-2.56	1.07	-6.75	0.72	-0.35	1.11	-90.57	0.80	-0.65	0.70	-0.65	0.71	-0.65	0.87	-2.10

9	Magnolol	0.67	-0.28	0.69	-0.27	0.81	-1.91	0.31	0.02	0.38	-0.10	0.46	-0.50	0.14	0.74	0.32	0.58	0.47	0.16
4	Maslinic acid (MA)	1.00	-22.77	0.85	-1.84	0.97	-13.80	0.44	0.29	0.87	-3.38	0.77	-1.78	0.10	0.77	0.35	0.56	0.52	0.38
70	Matrine ((+)-Matrine)	0.93	-7.29	0.99	-23.96	0.99	-70.75	0.68	0.23	0.98	-7.18	0.86	-3.93	0.72	-0.98	0.61	0.17	0.69	-0.96
72	Methyl-Hesperidin	0.89	-3.53	0.96	-5.58	0.94	-4.40	0.68	-0.14	0.33	-0.08	0.28	0.69	0.73	-1.01	0.44	0.47	0.55	0.07
63	Morin hydrate (Aurantica)	0.99	-451.48	0.31	0.56	0.13	0.78	0.63	0.10	0.27	0.14	0.18	0.77	0.65	-0.84	0.32	0.56	0.32	0.57
66	Myricetin (Cannabiscetin)	0.87	-2.48	0.90	-3.58	0.94	-7.67	0.68	-0.44	0.75	-1.04	0.72	-0.10	0.69	-0.90	0.75	-0.45	0.92	-5.03
76	Myricitrin (Myricitrine)	1.01	-22.49	1.01	-117.47	1.03	-11.90	0.73	-0.17	0.91	-3.19	0.83	-0.76	0.69	-1.06	0.77	-0.70	0.78	-1.05
104	Nalidixic acid (NegGram)	0.95	-15.09	1.12	-4.92	0.98	-22.89	0.84	-2.96	0.93	-3.96	0.99	-71.15	0.78	-1.89	0.96	-6.69	1.15	-3.88
126	Naringenin	0.96	-16.62	1.05	-13.65	1.04	-6.12	1.08	-3.48	1.18	-9.18	0.93	-3.42	0.90	-4.56	1.06	-4.48	1.06	-11.15
41	Naringin (Naringoside)	1.01	-19.85	1.11	-3.99	1.02	-16.92	0.71	0.29	0.93	-3.87	0.78	-1.28	0.71	-0.36	0.78	-0.71	0.86	-1.14
69	Naringin Dihydrochalcone (Naringin DC)	1.05	-5.90	1.14	-3.36	0.95	-2.97	1.15	-3.52	1.29	-8.29	0.93	-7.24	0.76	-0.95	0.87	-2.44	0.89	-2.10
96	Neohesperidin	1.03	-12.79	1.07	-4.08	1.00	-429.24	0.69	-0.17	0.89	-2.33	0.79	-1.13	0.74	-1.45	0.79	-0.38	0.90	-2.22
111	Neohesperidin dihydrochalcone (Nhdc)	0.91	-6.02	0.97	-8.23	0.91	-3.66	0.74	-0.78	0.87	-2.82	0.85	-0.60	0.79	-2.45	0.96	-7.53	1.19	-2.97
60	Nobiletin (Hexamethoxyflavone)	0.97	-44.59	1.12	-4.37	0.99	-36.45	0.62	-0.15	0.94	-4.56	1.08	-5.48	0.64	-0.72	0.87	-2.41	1.78	0.58
28	Oleanolic Acid (Caryophyllin)	1.05	-7.24	1.11	-1.88	1.01	-20.10	0.59	0.30	0.81	-1.49	0.76	0.24	0.58	0.08	0.67	0.20	0.74	-0.82
8	Oridonin (Isodonol)	1.10	-4.58	0.87	-1.46	0.99	-74.64	0.57	-1.84	0.29	-0.20	0.17	0.34	0.12	0.75	0.04	0.80	0.05	0.80
88	Orotic acid (6-Carboxyuracil)	1.03	-11.37	1.03	-22.85	1.00	-652.73	0.76	-0.23	0.87	-2.32	0.71	-0.51	0.76	-1.31	0.81	-0.44	0.85	-1.33
85	Osthole (Osthol)	0.98	-577.66	1.05	-11.49	0.87	-1.46	0.69	-1.27	0.86	-2.52	0.78	-0.24	0.70	-1.23	0.76	-1.09	1.09	-2.55
122	Oxymatrine (Matrine N-oxide)	1.07	-5.22	1.10	-3.93	0.99	-24.72	0.77	-0.34	0.95	-5.61	0.83	-1.32	0.86	-3.92	0.77	-0.64	0.97	-10.63
118	Paeonol (Peonol)	1.00	-22.79	1.14	-1.90	0.98	-23.68	0.79	-3.79	0.91	-4.06	0.88	-3.12	0.83	-3.57	0.91	-3.32	1.13	-3.60
62	Palmatine chloride	1.01	-18.26	1.05	-12.14	0.96	-4.97	0.79	-0.80	1.16	-14.90	1.20	-2.22	0.71	-0.78	1.55	-0.71	2.40	0.62
13	Parthenolide ((-)-Parthenolide)	1.31	-1.15	0.69	-0.59	0.68	-0.74	1.18	-6.47	0.60	-2.61	0.21	0.74	0.12	0.67	0.27	0.64	0.29	0.54
135	Phloretin (Dihydronaringenin)	0.92	-4.29	0.99	-25.49	0.99	-38.18	0.89	-5.51	0.99	-7.23	1.03	-9.16	0.95	-10.45	1.09	-3.52	1.43	-0.45
110	Phlorizin (Phloridzin)	1.01	-22.13	0.85	-1.69	1.00	-56.59	0.92	-12.80	1.23	-11.61	0.93	-6.11	0.84	-2.31	0.84	-0.98	0.92	-8.89
94	Piperine (1-Piperoylpiperidine)	0.89	-2.60	0.98	-12.11	0.98	-10.14	0.85	-2.71	0.90	-3.15	0.90	-2.01	0.74	-1.45	0.96	-7.35	1.40	-0.56
138	Polydatin(Piceid)	1.08	-4.22	1.07	-7.33	0.95	-3.70	1.19	-0.53	1.06	-39.83	0.84	-2.98	0.96	-17.80	0.85	-0.56	0.69	-0.07
127	Puerarin (Kakonein)	1.10	-4.27	0.94	-2.70	1.05	-5.72	0.98	-45.17	1.32	-8.52	0.95	-10.52	0.88	-5.86	0.91	-3.97	1.14	-2.13
51	Quercetin (Sophoretin)	1.00	-23.66	1.09	-6.17	0.86	-1.10	1.02	-51.19	1.01	-14.44	0.62	-0.59	0.56	-0.49	0.43	0.42	0.68	-0.10

33	Quercetin dihydrate (Sophoretin)	0.85	-1.46	0.93	-5.78	0.93	-4.24	0.77	-1.65	0.75	-1.53	0.71	-0.41	0.62	-0.06	0.64	0.21	0.73	-0.08
67	Resveratrol	1.09	-2.56	1.03	-33.03	1.02	-10.99	1.16	-1.45	1.08	-59.09	0.82	-2.09	0.76	-0.92	0.79	-0.86	0.81	-0.83
136	Rheochrysidin (Physcione)	0.91	-7.54	0.98	-11.42	0.96	-6.22	0.75	-0.32	1.02	-9.97	1.12	-3.96	0.98	-13.69	1.24	-2.79	1.44	-1.12
112	Rotundine	1.14	-1.09	1.13	-2.66	1.02	-24.89	1.00	-595.65	1.18	-13.64	0.89	-3.46	0.80	-2.55	0.88	-1.55	1.07	-5.78
18	Rutaecarpine (Rutecarpine)	0.95	-15.77	0.87	-2.18	0.92	-2.96	0.61	-2.70	0.98	-10.86	1.00	-130.25	0.56	0.40	0.92	-4.67	1.53	-0.32
115	Rutin (Rutoside)	0.87	-1.35	0.91	-1.83	0.97	-5.99	0.85	-2.61	0.85	-2.24	0.86	-0.34	0.87	-3.33	0.91	-3.12	1.25	-1.08
133	Salicin (Salicoside, Salicine)	1.06	-7.51	0.94	-5.96	0.98	-14.01	1.00	-596.77	1.06	-24.81	0.89	-2.98	0.86	-8.92	0.85	-0.78	0.91	-2.70
106	Salidroside (Rhodioloside)	0.87	-2.02	0.90	-2.18	1.04	-7.18	0.88	-2.38	1.13	-29.66	1.00	-152.55	0.83	-2.06	0.91	-2.32	1.18	-4.66
10	Sclareol	0.77	-0.95	0.75	-0.68	0.80	-0.63	0.21	0.64	0.42	-0.14	0.46	0.19	0.15	0.73	0.42	0.46	0.65	-0.79
17	Sclareolide (Norambreinolide)	1.05	-10.68	0.92	-3.94	0.95	-8.65	0.84	-9.20	0.88	-2.58	0.80	-0.22	0.27	0.46	0.53	0.24	0.68	-0.98
128	Sesamin (Fagarol)	1.08	-3.47	1.12	-3.65	0.96	-5.20	1.18	-2.01	1.14	-30.73	1.07	-7.19	0.90	-5.87	1.18	-12.73	0.93	-3.65
114	Shikimic acid (Shikimate)	0.94	-8.34	1.05	-6.68	1.07	-4.35	0.92	-4.83	0.97	-5.83	0.94	-8.55	0.81	-3.02	0.88	-1.70	1.14	-2.16
1	Shikonin	0.14	0.57	0.19	0.66	0.10	0.82	0.05	0.84	0.16	0.01	0.15	0.74	0.02	0.81	0.03	0.79	0.02	0.82
107	Silibinin (Silybin)	1.06	-5.13	1.03	-33.79	1.00	-40.73	1.00	-210.79	0.97	-6.21	0.89	-3.66	0.78	-2.13	0.90	-3.17	1.07	-8.33
123	Silymarin (Silybin B)	0.87	-2.51	0.91	-2.65	1.04	-7.48	0.78	-2.02	0.87	-2.74	0.81	-0.14	0.94	-3.99	1.02	-31.46	1.46	0.17
119	Sinomenine (Cucoline)	1.06	-5.01	1.08	-7.62	1.07	-5.16	1.13	-6.24	1.03	-16.56	0.86	-1.63	0.84	-3.57	0.89	-1.84	0.90	-3.25
47	Sodium Danshensu	0.97	-59.48	0.95	-6.16	1.00	-6751.80	0.77	-0.22	1.09	-347.11	0.95	-10.16	0.76	-0.43	1.15	-3.39	1.21	-0.83
84	Sophocarpine	1.07	-3.97	0.33	0.49	0.11	0.78	0.85	-1.44	0.32	-0.03	0.19	0.77	0.80	-1.23	0.29	0.66	0.35	0.48
105	Sorbitol (Glucitol)	1.07	-3.88	1.18	-2.00	1.08	-4.96	1.13	-4.95	1.48	-3.16	1.20	-3.15	0.85	-2.01	0.97	-18.87	1.06	-3.94
142	Synephrine (Oxedrine)	0.95	-14.34	1.00	-42.88	1.03	-10.16	0.85	-3.75	0.91	-2.95	0.81	-1.44	1.00	-379.01	0.92	-2.45	1.31	-1.01
108	Tangeretin (Tangeritin)	1.05	-7.73	1.13	-2.51	1.04	-6.21	1.04	-24.78	1.07	-50.82	0.91	-5.31	0.75	-2.17	0.82	-2.85	1.38	-1.19
89	Tanshinone I	0.92	-5.64	0.68	-0.86	0.95	-5.34	0.66	-0.39	0.57	-0.47	0.53	0.58	0.80	-1.32	0.37	0.03	0.62	0.23
52	Tanshinone IIA (Tanshinone B)	1.04	-7.67	0.92	-2.65	0.92	-3.64	0.96	-18.42	0.84	-2.56	0.52	0.33	0.72	-0.50	0.81	-1.63	1.00	-160.32
120	Taxifolin (Dihydroquercetin)	0.96	-18.04	0.97	-14.03	1.00	-39.76	0.87	-2.70	0.90	-2.75	0.89	-2.25	1.10	-3.64	0.97	-10.60	1.40	-0.28
141	Tetrahydropapaverine hydrochloride	1.05	-4.91	1.05	-7.63	0.97	-7.81	1.12	-3.60	1.03	-14.31	0.89	-3.64	1.00	-218.41	0.88	-2.31	0.99	-17.98
22	Tetrandrine (Fanchinine)	0.85	-2.50	0.53	0.29	0.47	-0.08	0.54	0.04	0.22	0.13	0.16	0.74	0.32	0.30	0.21	0.61	0.15	0.68
59	Triptolide	1.04	-4.47	0.22	0.62	0.19	0.75	0.97	-8.01	0.29	0.00	0.21	0.76	0.60	-0.70	0.25	0.64	0.42	0.47
125	Troxerutin	0.94	-10.42	1.00	-30.06	1.07	-3.01	0.92	-5.81	0.97	-5.27	0.88	-1.02	1.07	-4.48	0.95	-4.83	1.42	-0.73
3	Ursolic acid (Malol)	1.00	-53.89	1.06	-15.07	1.03	-11.70	0.69	-0.19	0.17	0.11	0.20	0.80	0.10	0.78	0.12	0.68	0.10	0.79

91	Vanillin	1.05	-3.69	1.05	-11.89	0.90	-2.70	1.16	-1.92	1.11	-83.81	0.84	-2.34	0.80	-1.36	0.99	-31.97	1.22	-0.92
44	Vanillylacetone	0.96	-16.18	1.01	-6096.09	1.01	-23.84	1.08	-9.72	1.07	-38.01	0.97	-14.26	0.80	-0.39	0.99	-25.86	1.19	-1.86
48	Xanthone (Genicide)	0.88	-2.73	1.02	-200.37	1.01	-28.07	0.89	-2.55	1.07	-35.15	1.01	-86.63	0.72	-0.44	0.85	-1.89	0.90	-1.64
39	Yohimbine hydrochloride (Antagonil)	0.95	-18.74	0.96	-9.73	0.95	-2.50	1.05	-18.57	1.05	-28.98	0.84	-1.13	0.65	-0.16	0.92	-3.80	1.08	-3.97
83	β-Sitosterol	0.91	-11.67	0.88	-8.33	0.73	-2.99	0.58	-0.84	0.68	-3.56	0.59	-3.19	0.60	-1.21	0.67	-2.65	0.72	-4.51

Supplementary Table 2. ATP bioluminescence values (normalized to control; mean ± standard deviation of 4 replicates) and z-factor derived from the ATP values in HMVP2 cells treated with UA in combination with each of the natural compounds listed at 20 µM dose and for 12, 24 or 48 hours.

	12 hours				24 hours				48 hours			
	ATP			z-factor	ATP			z-factor	ATP			z-factor
(-)-Epigallocatechin gallate	0.08	±	0.08	0.21	0.08	±	0.07	0.34	0.05	±	0.04	0.63
(+)-Usniacin (D-Usnic acid)	0.04	±	0.03	0.41	0.06	±	0.05	0.44	0.05	±	0.04	0.64
10-Hydroxycamptothecin	0.07	±	0.01	0.45	0.12	±	0.03	0.46	0.10	±	0.01	0.72
3-Indolebutyric acid (IBA)	0.08	±	0.07	0.23	0.12	±	0.11	0.18	0.07	±	0.05	0.59
4-Methylumbelliferone (4-MU)	0.05	±	0.03	0.38	0.11	±	0.08	0.28	0.09	±	0.09	0.48
5-Hydroxytryptophan (5-HTP)	0.18	±	0.03	0.29	0.15	±	0.04	0.40	0.11	±	0.02	0.67
Aesculin (Esculin)	0.02	±	0.01	0.46	0.06	±	0.05	0.43	0.05	±	0.03	0.66
Aloin (Barbaloin)	0.11	±	0.01	0.43	0.24	±	0.05	0.28	0.19	±	0.15	0.16
Ammonium Glycyrrhizinate (AMGZ)	0.28	±	0.03	0.19	0.17	±	0.02	0.45	0.23	±	0.22	-0.12
Amygdalin	0.02	±	0.03	0.41	0.05	±	0.03	0.48	0.18	±	0.21	-0.02
Andrographolide	0.05	±	0.05	0.34	0.14	±	0.09	0.22	0.39	±	0.20	-0.36
Apigenin	0.07	±	0.07	0.24	0.10	±	0.10	0.23	0.11	±	0.11	0.38
Apocynin (Acetovanillone)	0.11	±	0.02	0.39	0.17	±	0.06	0.33	0.11	±	0.02	0.70
Arbutin (Uva, p-Arbutin)	0.08	±	0.06	0.28	0.13	±	0.11	0.16	0.08	±	0.07	0.55
Artesunate	0.08	±	0.08	0.21	0.11	±	0.10	0.22	0.19	±	0.21	-0.05
Asiatic acid	0.04	±	0.03	0.39	0.14	±	0.14	0.06	0.06	±	0.05	0.61
Astragaloside A	0.13	±	0.02	0.36	0.11	±	0.02	0.48	0.13	±	0.03	0.66
Azomycin (2-Nitroimidazole)	0.06	±	0.04	0.33	0.08	±	0.07	0.34	0.04	±	0.03	0.69
Baicalein	0.10	±	0.08	0.18	0.10	±	0.09	0.25	0.09	±	0.06	0.57
Baicalin	0.06	±	0.05	0.32	0.12	±	0.11	0.18	0.08	±	0.08	0.51
Berberine Hydrochloride	0.07	±	0.06	0.29	0.11	±	0.10	0.22	0.16	±	0.17	0.14
Bergenin (Cuscutin)	0.13	±	0.10	0.09	0.15	±	0.13	0.07	0.13	±	0.13	0.29
Betulinic acid	0.11	±	0.02	0.39	0.33	±	0.03	0.27	0.12	±	0.02	0.70
Bilobalide	0.09	±	0.09	0.15	0.11	±	0.10	0.22	0.08	±	0.08	0.51
Biochanin A (4-Methylgenistein)	0.04	±	0.01	0.46	0.11	±	0.07	0.32	0.51	±	0.23	-0.83
Butylscopolamine bromide (Scopolamine butylbromide)	0.16	±	0.06	0.19	0.18	±	0.01	0.47	0.12	±	0.02	0.69
Caffeic acid	0.14	±	0.13	-0.04	0.13	±	0.12	0.15	0.15	±	0.13	0.28
Caffeic acid phenylethyl ester	0.08	±	0.02	0.41	0.23	±	0.05	0.30	0.11	±	0.02	0.69
Chlorogenic acid	0.24	±	0.22	-0.52	0.11	±	0.08	0.29	0.12	±	0.12	0.34
Chrysin	0.11	±	0.09	0.14	0.48	±	0.75	-4.05	0.06	±	0.05	0.59

Chrysophanic acid (Chrysophanol)	0.15	±	0.03	0.32	0.15	±	0.04	0.38	0.20	±	0.03	0.60
Cinchonidine	0.08	±	0.02	0.41	0.24	±	0.11	0.03	0.08	±	0.04	0.64
Corosolic acid (CA)	0.03	±	0.02	0.45	0.04	±	0.01	0.54	0.04	±	0.02	0.72
Cryptotanshinone	0.14	±	0.03	0.32	0.07	±	0.02	0.52	0.09	±	0.04	0.62
Curcumin	0.03	±	0.01	0.46	0.05	±	0.02	0.52	0.06	±	0.02	0.71
Curcumol	0.14	±	0.05	0.27	0.20	±	0.04	0.34	0.22	±	0.15	0.13
Cyclocytidine HCl	0.06	±	0.04	0.35	0.07	±	0.05	0.42	0.04	±	0.02	0.71
Cyclosporin A (Cyclosporine A)	0.20	±	0.10	0.03	0.08	±	0.01	0.54	0.18	±	0.04	0.60
Cytisine (Baphitoxine, Sophorine)	0.15	±	0.02	0.35	0.18	±	0.01	0.48	0.16	±	0.08	0.46
Dihydroartemisinin (DHA)	0.06	±	0.02	0.43	0.11	±	0.05	0.40	0.09	±	0.03	0.65
Dihydromyricetin (Ampeloptin)	0.15	±	0.07	0.17	0.18	±	0.05	0.35	0.15	±	0.02	0.67
Dioscin (Collettiside III)	0.01	±	0.00	0.50	0.02	±	0.00	0.59	0.03	±	0.01	0.75
Diosmetin (Luteolin 4-methyl ether)	0.10	±	0.04	0.32	0.16	±	0.03	0.42	0.22	±	0.20	-0.05
Diosmin	0.10	±	0.01	0.42	0.20	±	0.02	0.42	0.20	±	0.11	0.32
DL-Carnitine hydrochloride	0.11	±	0.05	0.27	0.10	±	0.03	0.47	0.41	±	0.31	-0.94
D-Mannitol (Osmitrol)	0.12	±	0.02	0.37	0.23	±	0.04	0.34	0.17	±	0.03	0.63
Emodin	0.08	±	0.02	0.40	0.18	±	0.09	0.20	0.33	±	0.19	-0.17
Enoxolone (Glycyrrhetin)	0.08	±	0.03	0.37	0.15	±	0.08	0.27	0.19	±	0.19	0.04
Epi-Corosolic acid (ECA)	0.09	±	0.05	0.30	0.14	±	0.02	0.48	0.21	±	0.02	0.66
Epi-Maslinic acid (EMA)	0.05	±	0.01	0.46	0.14	±	0.02	0.46	0.18	±	0.03	0.62
Epi-Ursolic acid (EUA)	0.11	±	0.03	0.34	0.11	±	0.02	0.47	0.33	±	0.24	-0.42
Fisetin (Fustel)	0.09	±	0.02	0.40	0.16	±	0.01	0.48	0.16	±	0.05	0.55
Formononetin (Formononetol)	0.06	±	0.02	0.42	0.10	±	0.04	0.43	0.23	±	0.29	-0.41
Fumalic acid (Ferulic acid)	0.12	±	0.03	0.34	0.22	±	0.01	0.45	0.17	±	0.07	0.48
Gastrodin (Gastrodine)	0.16	±	0.08	0.12	0.19	±	0.03	0.39	0.13	±	0.01	0.71
Genistein	0.12	±	0.03	0.33	0.18	±	0.04	0.38	0.13	±	0.05	0.58
Glycyrrhizic acid	0.12	±	0.05	0.28	0.14	±	0.02	0.47	0.09	±	0.01	0.72
Gossypol	0.04	±	0.01	0.47	0.21	±	0.03	0.39	0.06	±	0.02	0.69
Gramine	0.07	±	0.03	0.37	0.13	±	0.02	0.47	0.15	±	0.10	0.38
Guanosine	0.09	±	0.03	0.36	0.11	±	0.04	0.41	0.13	±	0.03	0.66
Gynostemma Extract	0.13	±	0.04	0.30	0.10	±	0.04	0.43	0.31	±	0.19	-0.15
Hematoxylin (Hydroxybrazilin)	0.11	±	0.04	0.32	0.18	±	0.03	0.43	0.08	±	0.01	0.74
Hesperetin	0.12	±	0.04	0.30	0.17	±	0.03	0.43	0.10	±	0.03	0.67
Hesperidin	0.12	±	0.03	0.34	0.20	±	0.06	0.30	0.21	±	0.15	0.15

Honokiol	0.05	±	0.01	0.44	0.07	±	0.01	0.54	0.18	±	0.24	-0.15
Hordenine	0.07	±	0.02	0.39	0.15	±	0.05	0.36	0.14	±	0.09	0.44
Hyodeoxycholic acid (HDCA)	0.29	±	0.08	-0.01	0.18	±	0.04	0.38	0.20	±	0.10	0.37
Hypoxanthine	0.09	±	0.03	0.37	0.14	±	0.03	0.44	0.16	±	0.06	0.52
Icariin	0.17	±	0.05	0.23	0.16	±	0.04	0.37	0.24	±	0.18	0.01
Indirubin	0.05	±	0.01	0.46	0.12	±	0.06	0.35	0.08	±	0.01	0.73
Indole-3-carbinol	0.25	±	0.06	0.09	0.23	±	0.04	0.34	0.22	±	0.02	0.66
Inosine	0.14	±	0.06	0.22	0.19	±	0.04	0.38	0.20	±	0.13	0.25
Ipriflavone	0.08	±	0.02	0.41	0.12	±	0.01	0.51	0.12	±	0.04	0.61
Isoliquiritigenin	0.13	±	0.04	0.29	0.20	±	0.05	0.34	0.46	±	0.16	-0.26
Kaempferol	0.08	±	0.02	0.41	0.13	±	0.02	0.47	0.12	±	0.02	0.68
Kinetin (6-Furfuryladenine)	0.27	±	0.19	-0.45	0.24	±	0.04	0.33	0.17	±	0.03	0.62
L (+)-Rhamnose Monohydrate	0.10	±	0.01	0.42	0.15	±	0.02	0.47	0.14	±	0.07	0.50
Lappaconite Hydrobromide	0.18	±	0.06	0.20	0.19	±	0.03	0.42	0.13	±	0.02	0.69
Limonin	0.16	±	0.10	0.07	0.15	±	0.02	0.47	0.13	±	0.03	0.63
Luteolin	0.31	±	0.37	-1.31	0.12	±	0.04	0.43	0.18	±	0.04	0.59
Magnolol	0.05	±	0.01	0.46	0.09	±	0.01	0.53	0.13	±	0.14	0.27
Maslinic acid (MA)	0.04	±	0.01	0.47	0.15	±	0.02	0.48	0.19	±	0.02	0.66
Matrine ((+)-Matrine)	0.11	±	0.03	0.36	0.14	±	0.03	0.45	0.30	±	0.31	-0.66
Methyl-Hesperidin	0.20	±	0.16	-0.20	0.35	±	0.06	0.11	0.27	±	0.14	0.14
Morin hydrate (Aurantica)	0.26	±	0.04	0.16	0.17	±	0.02	0.44	0.17	±	0.04	0.60
Myricetin (Cannabiscetin)	0.14	±	0.03	0.31	0.17	±	0.03	0.42	0.14	±	0.04	0.61
Myricitrin (Myricitrine)	0.09	±	0.02	0.38	0.15	±	0.03	0.45	0.20	±	0.19	0.03
Nalidixic acid (NegGram)	0.15	±	0.04	0.30	0.20	±	0.02	0.45	0.14	±	0.04	0.60
Naringenin	0.27	±	0.10	-0.07	0.24	±	0.05	0.27	0.14	±	0.03	0.66
Naringin (Naringoside)	0.19	±	0.05	0.22	0.19	±	0.02	0.45	0.14	±	0.04	0.63
Naringin Dihydrochalcone (Naringin DC)	0.17	±	0.05	0.21	0.24	±	0.05	0.30	0.14	±	0.02	0.66
Neohesperidin	0.16	±	0.11	0.03	0.15	±	0.03	0.42	0.19	±	0.11	0.33
Neohesperidin dihydrochalcone (Nhdc)	0.10	±	0.02	0.40	0.17	±	0.04	0.38	0.13	±	0.04	0.61
Nobiletin (Hexamethoxyflavone)	0.08	±	0.03	0.38	0.17	±	0.02	0.46	0.17	±	0.04	0.61
Oleanolic Acid (Caryophyllin)	0.04	±	0.01	0.45	0.07	±	0.02	0.52	0.10	±	0.04	0.61
Oridonin (Isodonol)	0.05	±	0.01	0.46	0.02	±	0.00	0.60	0.02	±	0.00	0.77
Orotic acid (6-Carboxyuracil)	0.24	±	0.03	0.23	0.21	±	0.01	0.45	0.15	±	0.10	0.41
Osthole (Osthol)	0.15	±	0.04	0.27	0.16	±	0.05	0.34	0.32	±	0.21	-0.26

Oxymatrine (Matrine N-oxide)	0.20	±	0.07	0.14	0.21	±	0.08	0.21	0.23	±	0.09	0.36
Paeonol (Peonol)	0.22	±	0.06	0.14	0.21	±	0.04	0.35	0.16	±	0.04	0.62
Palmatine chloride	0.24	±	0.08	0.06	0.23	±	0.07	0.20	0.43	±	0.04	0.43
Parthenolide ((-)-Parthenolide)	0.05	±	0.01	0.46	0.21	±	0.01	0.45	0.15	±	0.05	0.56
Phloretin (Dihydronaringenin)	0.25	±	0.09	-0.02	0.23	±	0.03	0.38	0.16	±	0.04	0.59
Phlorizin (Phloridzin)	0.20	±	0.03	0.29	0.22	±	0.04	0.34	0.19	±	0.07	0.48
Piperine (1-Piperoylpiperidine)	0.15	±	0.04	0.27	0.18	±	0.03	0.39	0.16	±	0.06	0.52
Polydatin(Piceid)	0.16	±	0.04	0.26	0.22	±	0.05	0.29	0.16	±	0.03	0.65
Puerarin (Kakonein)	0.18	±	0.03	0.29	0.21	±	0.05	0.31	0.22	±	0.07	0.44
Quercetin (Sophoretin)	0.12	±	0.01	0.40	0.23	±	0.03	0.39	0.15	±	0.04	0.59
Quercetin dihydrate (Sophoretin)	0.17	±	0.02	0.33	0.26	±	0.02	0.40	0.24	±	0.13	0.19
Resveratrol	0.15	±	0.03	0.31	0.27	±	0.05	0.24	0.28	±	0.23	-0.25
Rheochrysidin (Physcione)	0.08	±	0.02	0.41	0.17	±	0.06	0.31	0.10	±	0.02	0.68
Rotundine	0.12	±	0.06	0.25	0.11	±	0.02	0.49	0.09	±	0.01	0.72
Rutaecarpine (Rutecarpine)	0.11	±	0.01	0.42	0.12	±	0.01	0.50	0.29	±	0.30	-0.58
Rutin (Rutoside)	0.18	±	0.03	0.28	0.23	±	0.07	0.23	0.45	±	0.14	-0.16
Salicin (Salicoside, Salicine)	0.19	±	0.08	0.10	0.21	±	0.03	0.39	0.15	±	0.03	0.64
Salidroside (Rhodioloside)	0.19	±	0.02	0.33	0.23	±	0.07	0.23	0.14	±	0.02	0.69
Sclareol	0.05	±	0.01	0.45	0.13	±	0.03	0.44	0.06	±	0.03	0.68
Sclareolide (Norambreinolide)	0.07	±	0.01	0.43	0.13	±	0.03	0.44	0.18	±	0.24	-0.13
Sesamin (Fagarol)	0.12	±	0.04	0.30	0.18	±	0.04	0.38	0.23	±	0.16	0.11
Shikimic acid (Shikimate)	0.11	±	0.03	0.35	0.19	±	0.03	0.40	0.12	±	0.03	0.65
Shikonin	0.01	±	0.00	0.50	0.01	±	0.00	0.60	0.01	±	0.00	0.77
Silibinin (Silybin)	0.25	±	0.11	-0.08	0.20	±	0.01	0.45	0.16	±	0.02	0.66
Silymarin (Silybin B)	0.22	±	0.04	0.23	0.21	±	0.03	0.40	0.14	±	0.04	0.61
Sinomenine (Cucoline)	0.18	±	0.03	0.28	0.23	±	0.03	0.38	0.18	±	0.05	0.55
Sodium Danshensu	0.19	±	0.02	0.32	0.24	±	0.06	0.25	0.19	±	0.10	0.36
Sophocarpine	0.12	±	0.04	0.30	0.18	±	0.03	0.40	0.14	±	0.02	0.67
Sorbitol (Glucitol)	0.16	±	0.04	0.27	0.23	±	0.07	0.22	0.13	±	0.03	0.65
Synephrine (Oxedrine)	0.20	±	0.06	0.18	0.38	±	0.13	-0.28	0.22	±	0.07	0.46
Tangeretin (Tangeritin)	0.13	±	0.06	0.22	0.24	±	0.06	0.25	0.20	±	0.13	0.23
Tanshinone I	0.07	±	0.01	0.43	0.09	±	0.01	0.53	0.05	±	0.01	0.73
Tanshinone IIA (Tanshinone B)	0.34	±	0.11	-0.23	0.29	±	0.11	-0.01	0.16	±	0.08	0.47
Taxifolin (Dihydroquercetin)	0.26	±	0.05	0.13	0.29	±	0.10	0.04	0.28	±	0.07	0.43

Tetrahydropapaverine hydrochloride	0.27	±	0.08	-0.02	0.20	±	0.03	0.39	0.24	±	0.08	0.40
Tetrandrine (Fanchinine)	0.04	±	0.01	0.45	0.11	±	0.06	0.36	0.23	±	0.29	-0.43
Triptolide	0.12	±	0.02	0.38	0.20	±	0.03	0.37	0.12	±	0.01	0.70
Troxerutin	0.23	±	0.04	0.23	0.21	±	0.02	0.43	0.14	±	0.05	0.59
Ursolic acid (Malol)	0.07	±	0.10	0.14	0.03	±	0.01	0.57	0.03	±	0.00	0.76
Vanillin	0.09	±	0.02	0.40	0.18	±	0.02	0.44	0.11	±	0.02	0.67
Vanillylacetone	0.21	±	0.03	0.26	0.23	±	0.03	0.37	0.17	±	0.05	0.55
Xanthone (Genicide)	0.12	±	0.03	0.34	0.19	±	0.02	0.45	0.13	±	0.02	0.68
Yohimbine hydrochloride (Antagonil)	0.16	±	0.03	0.30	0.23	±	0.03	0.37	0.13	±	0.03	0.66
β-Sitosterol	0.12	±	0.10	0.09	0.13	±	0.12	0.14	0.09	±	0.09	0.46

Supplementary Table 3. ATP bioluminescence values (normalized to control; mean ± standard deviation of 4 replicates) and z-factor in DU145, PC3 and C4-2B human PCa cells treated with UA in combination with each of the natural compounds listed at 20 µM for 12 hours.

	DU145				PC3				C4-2B			
	ATP			z-factor	ATP			z-factor	ATP			z-factor
(-)-Epigallocatechin gallate	0.60	±	0.11	-0.33	0.58	±	0.18	-1.78	0.31	±	0.27	-0.76
(+)-Usniacin (D-Usnic acid)	0.28	±	0.15	0.10	0.82	±	0.07	-3.53	0.13	±	0.10	0.18
10-Hydroxycamptothecin	0.89	±	0.21	-6.47	0.81	±	0.10	-3.76	0.36	±	0.02	0.27
3-Indolebutyric acid (IBA)	0.65	±	0.06	-0.14	0.80	±	0.23	-5.40	0.39	±	0.32	-1.21
4-Methylumbelliferone (4-MU)	0.69	±	0.21	-1.71	0.89	±	0.22	-10.53	0.30	±	0.26	-0.68
5-hydroxytryptophan (5-HTP)	0.70	±	0.09	-0.60	0.91	±	0.19	-11.62	0.52	±	0.09	-0.42
Aesculin (Esculin)	0.59	±	0.17	-0.74	0.60	±	0.42	-3.67	0.21	±	0.20	-0.28
Aloin (Barbaloin)	0.77	±	0.08	-0.98	0.84	±	0.23	-7.24	0.73	±	0.15	-2.08
Ammonium Glycyrrhizinate (AMGZ)	0.92	±	0.10	-5.49	0.93	±	0.17	-14.84	0.68	±	0.14	-1.57
Amygdalin	0.04	±	0.02	0.73	0.05	±	0.01	0.34	0.06	±	0.05	0.42
Andrographolide	0.80	±	0.24	-3.66	0.34	±	0.32	-1.36	0.30	±	0.27	-0.71
Apigenin	0.81	±	0.28	-4.53	1.34	±	0.02	-1.00	0.32	±	0.21	-0.53
Apocynin (Acetovanillone)	0.89	±	0.25	-8.07	0.81	±	0.04	-2.78	0.46	±	0.06	-0.04
Arbutin (Uva, p-Arbutin)	0.98	±	0.19	-32.79	0.78	±	0.42	-7.46	0.37	±	0.33	-1.19
Artesunate	0.74	±	0.29	-3.15	1.01	±	0.25	-137.41	0.26	±	0.21	-0.39
Asiatic acid	0.35	±	0.24	-0.41	0.31	±	0.22	-0.80	0.12	±	0.09	0.23
Astragaloside A	0.89	±	0.29	-8.90	0.90	±	0.10	-7.46	0.42	±	0.06	-0.01
Azomycin (2-Nitroimidazole)	0.81	±	0.43	-6.95	1.15	±	0.19	-7.15	0.29	±	0.26	-0.65
Baicalein	0.89	±	0.20	-6.30	0.81	±	0.38	-8.34	0.30	±	0.26	-0.66
Baicalin	0.83	±	0.37	-6.78	1.18	±	0.06	-3.42	0.29	±	0.26	-0.67
Berberine Hydrochloride	1.10	±	0.35	-11.94	0.98	±	0.07	-31.23	0.29	±	0.24	-0.56
Bergenin (Cuscutin)	0.93	±	0.41	-20.54	0.79	±	0.33	-6.60	0.51	±	0.27	-1.45
Betulinic acid	0.12	±	0.03	0.65	0.20	±	0.09	-0.07	0.37	±	0.05	0.14
Bilobalide	1.04	±	0.56	-50.05	1.27	±	0.17	-3.06	0.35	±	0.22	-0.60
Biochanin A (4-Methylgenistein)	0.59	±	0.06	0.02	0.47	±	0.14	-0.94	0.48	±	0.11	-0.40
Butylscopolamine bromide (Scopolamine butylbromide)	0.81	±	0.05	-0.85	0.53	±	0.12	-1.00	0.47	±	0.09	-0.23
Caffeic acid	1.20	±	0.59	-8.73	0.92	±	0.42	-23.31	0.44	±	0.23	-0.98
Caffeic acid phenylethyl ester	0.08	±	0.00	0.76	0.11	±	0.03	0.23	0.39	±	0.09	-0.12
Chlorogenic acid	1.12	±	0.38	-10.43	1.21	±	0.17	-4.23	0.31	±	0.16	-0.27
Chrysin	1.34	±	0.32	-2.45	0.77	±	0.44	-7.49	0.60	±	0.21	-1.61

Chrysophanic acid (Chrysophanol)	0.90	±	0.30	-10.68	1.01	±	0.11	-77.75	0.64	±	0.09	-0.86
Cinchonidine	0.63	±	0.12	-0.59	0.85	±	0.20	-6.71	0.34	±	0.02	0.33
Corosolic acid (CA)	0.11	±	0.03	0.68	0.12	±	0.08	0.06	0.11	±	0.03	0.45
Cryptotanshinone	0.12	±	0.01	0.71	0.28	±	0.06	-0.08	0.13	±	0.01	0.50
Curcumin	0.10	±	0.02	0.70	0.20	±	0.11	-0.16	0.25	±	0.11	0.05
Curcumol	0.49	±	0.06	0.24	0.40	±	0.08	-0.40	0.38	±	0.14	-0.33
Cyclocytidine HCl	0.52	±	0.25	-1.04	0.71	±	0.08	-1.89	0.19	±	0.18	-0.17
Cyclosporin A (Cyclosporine A)	0.68	±	0.14	-0.96	0.64	±	0.17	-2.12	0.19	±	0.05	0.32
Cytisine (Baphitoxine, Sophorine)	0.84	±	0.18	-3.84	0.77	±	0.12	-3.20	0.59	±	0.12	-0.84
Dihydroartemisinin (DHA)	0.74	±	0.10	-0.98	0.77	±	0.20	-4.07	0.30	±	0.08	0.09
Dihydromyricetin (Ampeloptin)	0.58	±	0.06	0.06	0.68	±	0.20	-2.80	0.52	±	0.19	-1.06
Dioscin (Collettiside III)	0.03	±	0.00	0.77	0.03	±	0.00	0.37	0.05	±	0.01	0.57
Diosmetin (Luteolin 4-methyl ether)	0.99	±	0.04	-41.57	0.88	±	0.22	-9.05	0.58	±	0.13	-0.89
Diosmin	0.88	±	0.16	-4.70	0.84	±	0.20	-6.46	0.54	±	0.05	-0.17
DL-Carnitine hydrochloride	0.84	±	0.08	-1.72	0.92	±	0.10	-10.04	0.34	±	0.08	0.04
D-Mannitol (Osmitrol)	0.87	±	0.09	-2.88	0.76	±	0.17	-3.55	0.85	±	0.36	-8.80
Emodin	0.50	±	0.08	0.14	0.55	±	0.12	-1.13	0.41	±	0.05	0.05
Enoxolone (Glycyrrhetin)	0.74	±	0.07	-0.54	1.01	±	0.14	-112.42	0.35	±	0.08	0.04
Epi-Corosolic acid (ECA)	0.59	±	0.07	-0.06	0.51	±	0.07	-0.65	0.56	±	0.11	-0.65
Epi-Maslinic acid (EMA)	0.42	±	0.12	0.03	0.39	±	0.11	-0.52	0.44	±	0.09	-0.19
Epi-Ursolic acid (EUA)	0.57	±	0.10	-0.20	0.64	±	0.25	-2.75	0.51	±	0.10	-0.44
Fisetin (Fustel)	1.10	±	0.15	-5.45	1.08	±	0.09	-10.29	0.56	±	0.10	-0.56
Formononetin (Formononetol)	0.87	±	0.08	-2.45	1.00	±	0.19	-392.35	0.35	±	0.12	-0.14
Fumalic acid (Ferulic acid)	1.22	±	0.44	-6.07	1.01	±	0.09	-107.62	0.56	±	0.11	-0.65
Gastrodin (Gastrodine)	0.90	±	0.05	-2.72	0.91	±	0.15	-10.22	0.56	±	0.15	-0.94
Genistein	0.69	±	0.14	-1.07	0.92	±	0.21	-14.89	0.38	±	0.13	-0.29
Glycyrrhizic acid	1.18	±	0.35	-5.98	1.08	±	0.16	-12.58	0.49	±	0.11	-0.41
Gossypol	0.57	±	0.48	-2.83	0.20	±	0.03	0.14	0.15	±	0.06	0.33
Gramine	1.00	±	0.33	-252.82	0.96	±	0.16	-27.11	0.40	±	0.12	-0.25
Guanosine	0.92	±	0.26	-11.19	0.77	±	0.10	-2.81	0.41	±	0.05	0.06
Gynostemma Extract	1.29	±	0.47	-4.57	1.01	±	0.15	-71.20	0.49	±	0.05	-0.06
Hematoxylin (Hydroxybrazilin)	0.76	±	0.07	-0.73	0.58	±	0.11	-1.25	0.61	±	0.16	-1.23
Hesperetin	1.24	±	0.25	-3.03	1.22	±	0.20	-4.38	0.52	±	0.16	-0.82
Hesperidin	1.37	±	0.43	-3.00	1.21	±	0.10	-3.17	0.48	±	0.12	-0.45

Honokiol	0.74	±	0.76	-8.42	0.13	±	0.03	0.22	0.17	±	0.02	0.44
Hordenine	0.80	±	0.06	-0.96	0.91	±	0.11	-9.72	0.43	±	0.07	-0.05
Hyodeoxycholic acid (HDCA)	1.55	±	0.38	-1.43	1.26	±	0.16	-3.09	0.62	±	0.14	-1.19
Hypoxanthine	0.93	±	0.10	-6.59	0.65	±	0.09	-1.45	0.26	±	0.08	0.15
Icariin	1.57	±	0.32	-1.07	0.97	±	0.08	-30.71	0.54	±	0.14	-0.81
Indirubin	0.57	±	0.05	0.14	0.37	±	0.04	-0.14	0.36	±	0.04	0.19
Indole-3-carbinol	1.39	±	0.39	-2.51	1.32	±	0.15	-2.30	0.64	±	0.08	-0.80
Inosine	1.13	±	0.08	-2.52	0.86	±	0.10	-5.47	0.49	±	0.32	-1.64
Ipriflavone	0.34	±	0.02	0.57	0.36	±	0.07	-0.26	0.34	±	0.12	-0.14
Isoliquiritigenin	0.86	±	0.35	-8.30	0.71	±	0.17	-2.81	0.48	±	0.05	-0.03
Kaempferol	0.88	±	0.33	-9.00	0.96	±	0.06	-20.96	0.59	±	0.22	-1.57
Kinetin (6-Furfuryladenine)	0.95	±	0.16	-12.92	0.95	±	0.19	-20.89	0.59	±	0.05	-0.33
L (+)-Rhamnose Monohydrate	0.77	±	0.28	-3.56	0.87	±	0.06	-5.05	0.62	±	0.15	-1.23
Lappaconite Hydrobromide	0.82	±	0.10	-1.87	0.89	±	0.18	-9.12	0.53	±	0.04	-0.12
Limonin	0.98	±	0.18	-44.06	1.00	±	0.26	-312.72	0.51	±	0.08	-0.34
Luteolin	0.89	±	0.29	-8.87	1.15	±	0.17	-6.40	0.80	±	0.38	-6.63
Magnolol	0.10	±	0.02	0.72	0.09	±	0.01	0.31	0.21	±	0.04	0.36
Maslinic acid (MA)	0.10	±	0.03	0.67	0.15	±	0.09	-0.01	0.14	±	0.04	0.40
Matrine ((+)-Matrine)	0.77	±	0.14	-1.73	0.80	±	0.08	-3.15	0.58	±	0.20	-1.37
Methyl-Hesperidin	1.00	±	0.16	-161.79	0.99	±	0.22	-108.74	0.57	±	0.10	-0.63
Morin hydrate (Aurantica)	0.94	±	0.13	-9.31	0.99	±	0.04	-55.40	0.88	±	0.33	-10.19
Myricetin (Cannabiscetin)	0.97	±	0.16	-25.02	0.96	±	0.22	-29.05	0.47	±	0.03	0.11
Myricitrin (Myricitrine)	0.88	±	0.09	-3.03	0.91	±	0.10	-9.39	0.75	±	0.34	-4.73
Nalidixic acid (NegGram)	1.14	±	0.31	-7.08	1.05	±	0.18	-21.97	0.62	±	0.04	-0.42
Naringenin	0.55	±	0.11	-0.17	0.74	±	0.17	-3.22	0.50	±	0.12	-0.51
Naringin (Naringoside)	0.91	±	0.08	-3.89	0.92	±	0.10	-10.65	0.75	±	0.29	-4.07
Naringin Dihydrochalcone (Naringin DC)	1.04	±	0.15	-15.93	0.75	±	0.25	-4.53	0.94	±	0.37	-22.59
Neohesperidin	0.95	±	0.04	-5.29	1.03	±	0.13	-29.77	0.86	±	0.29	-7.89
Neohesperidin dihydrochalcone (Nhdc)	1.23	±	0.30	-3.72	1.06	±	0.18	-17.48	0.57	±	0.03	-0.16
Nobiletin (Hexamethoxyflavone)	1.26	±	0.39	-4.30	1.01	±	0.14	-121.76	0.58	±	0.15	-1.00
Oleanolic Acid (Caryophyllin)	0.84	±	0.08	-1.83	0.78	±	0.19	-4.32	0.38	±	0.16	-0.44
Oridonin (Isodonol)	1.69	±	0.35	-0.81	0.87	±	0.05	-4.98	0.21	±	0.07	0.24
Orotic acid (6-Carboxyuracil)	1.06	±	0.06	-5.64	1.17	±	0.19	-5.72	0.87	±	0.28	-8.41
Osthole (Osthol)	1.41	±	0.35	-2.09	0.86	±	0.13	-5.94	0.78	±	0.26	-4.42

Oxymatrine (Matrine N-oxide)	0.97	±	0.12	-15.60	1.00	±	0.08	-177.57	1.15	±	0.53	-12.64
Paeonol (Peonol)	1.57	±	0.14	-0.12	1.08	±	0.08	-9.66	0.74	±	0.55	-7.06
Palmatine chloride	0.78	±	0.30	-4.12	0.90	±	0.31	-13.93	0.68	±	0.06	-0.80
Parthenolide ((-)-Parthenolide)	0.34	±	0.08	0.30	0.40	±	0.10	-0.47	0.20	±	0.04	0.34
Phloretin (Dihydronaringenin)	0.92	±	0.23	-9.91	1.06	±	0.31	-23.14	0.65	±	0.17	-1.60
Phlorizin (Phloridzin)	0.79	±	0.12	-1.68	1.07	±	0.12	-12.90	0.75	±	0.27	-3.93
Piperine (1-Piperoylpiperidine)	0.83	±	0.24	-4.50	1.05	±	0.18	-23.92	0.67	±	0.26	-2.57
Polydatin(Piceid)	1.12	±	0.14	-4.04	1.11	±	0.25	-10.82	0.51	±	0.06	-0.18
Puerarin (Kakonein)	0.78	±	0.16	-2.15	1.20	±	0.12	-3.76	0.71	±	0.28	-3.23
Quercetin (Sophoretin)	1.31	±	0.28	-2.37	0.73	±	0.15	-3.00	0.86	±	0.36	-9.64
Quercetin dihydrate (Sophoretin)	0.95	±	0.18	-15.54	1.10	±	0.24	-12.38	0.67	±	0.16	-1.66
Resveratrol	1.05	±	0.29	-22.09	0.94	±	0.20	-20.47	0.70	±	0.09	-1.23
Rheochrysidin (Physcione)	0.60	±	0.28	-1.61	0.63	±	0.25	-2.63	0.33	±	0.01	0.36
Rotundine	0.75	±	0.08	-0.86	0.99	±	0.41	-191.41	0.34	±	0.15	-0.30
Rutaecarpine (Rutecarpine)	0.19	±	0.04	0.60	0.47	±	0.35	-2.10	0.40	±	0.26	-0.97
Rutin (Rutoside)	0.94	±	0.20	-12.60	0.96	±	0.20	-31.21	0.63	±	0.16	-1.42
Salicin (Salicoside, Salicine)	0.88	±	0.14	-4.22	0.99	±	0.29	-237.97	0.68	±	0.19	-1.99
Salidroside (Rhodioloside)	0.52	±	0.08	0.08	0.88	±	0.25	-10.37	0.52	±	0.17	-0.86
Sclareol	0.08	±	0.02	0.72	0.11	±	0.01	0.30	0.23	±	0.09	0.13
Sclareolide (Norambreinolide)	0.15	±	0.04	0.61	0.35	±	0.15	-0.62	0.58	±	0.32	-2.23
Sesamin (Fagarol)	0.94	±	0.25	-15.54	0.93	±	0.17	-14.41	0.75	±	0.14	-2.25
Shikimic acid (Shikimate)	1.03	±	0.12	-20.28	0.99	±	0.17	-89.88	0.84	±	0.30	-6.91
Shikonin	0.01	±	0.00	0.78	0.04	±	0.01	0.33	0.02	±	0.01	0.58
Silibinin (Silybin)	0.90	±	0.05	-2.53	1.12	±	0.33	-12.00	0.98	±	0.40	-72.57
Silymarin (Silybin B)	1.03	±	0.18	-26.66	1.10	±	0.20	-11.11	0.89	±	0.27	-9.66
Sinomenine (Cucoline)	0.96	±	0.10	-11.01	1.11	±	0.25	-11.59	0.81	±	0.35	-6.55
Sodium Danshensu	0.83	±	0.31	-5.76	0.93	±	0.21	-15.45	0.65	±	0.03	-0.44
Sophocarpine	0.81	±	0.05	-0.95	0.65	±	0.28	-3.17	0.39	±	0.14	-0.33
Sorbitol (Glucitol)	1.23	±	0.32	-4.01	0.70	±	0.09	-1.87	0.96	±	0.73	-69.12
Synephrine (Oxedrine)	1.01	±	0.20	-61.43	1.08	±	0.17	-13.44	0.83	±	0.61	-12.16
Tangeretin (Tangeritin)	0.87	±	0.13	-3.69	1.02	±	0.40	-87.37	0.83	±	0.30	-6.62
Tanshinone I	0.71	±	0.14	-1.13	0.61	±	0.19	-2.04	0.27	±	0.03	0.33
Tanshinone IIA (Tanshinone B)	0.40	±	0.10	0.14	0.85	±	0.38	-10.62	0.51	±	0.11	-0.49
Taxifolin (Dihydroquercetin)	1.13	±	0.13	-3.64	0.98	±	0.14	-45.82	0.99	±	0.30	-193.18

Tetrahydropapaverine hydrochloride	1.02	±	0.15	-27.31	1.07	±	0.31	-22.39	0.93	±	0.51	-28.41
Tetrandrine (Fanchinine)	0.36	±	0.10	0.21	0.26	±	0.10	-0.21	0.17	±	0.03	0.42
Triptolide	1.06	±	0.09	-6.72	0.92	±	0.10	-10.39	0.62	±	0.28	-2.28
Troxerutin	1.15	±	0.31	-6.48	1.02	±	0.20	-53.06	1.03	±	0.62	-82.38
Ursolic acid (Malol)	0.31	±	0.10	0.26	0.57	±	0.43	-3.42	0.07	±	0.02	0.52
Vanillin	1.12	±	0.24	-6.98	0.87	±	0.13	-6.66	0.53	±	0.08	-0.34
Vanillylacetone	0.83	±	0.11	-2.26	0.93	±	0.21	-17.61	0.81	±	0.17	-3.74
Xanthone (Genicide)	0.58	±	0.13	-0.43	0.92	±	0.19	-13.00	0.57	±	0.07	-0.43
Yohimbine hydrochloride (Antagonil)	0.76	±	0.09	-1.00	0.89	±	0.21	-9.98	0.75	±	0.14	-2.19
β-Sitosterol	1.10	±	0.56	-17.49	0.93	±	0.43	-24.53	0.45	±	0.25	-1.08

Supplementary Table 4. Fold change (first row) and p-value (italic on second row) of intracellular lipids (grouped by sub-class) following treatment with CUR, UA and RES, either alone or in combination of 2 compounds (compared to control). The total concentration of all lipids in each sub-class was used for calculation of fold change and p-values. Statistical significance is based on student t-test with p<0.05 being significant.

Lipid subclass	Lipid class	CUR	UA	RES	CUR+UA	RES+UA	CUR+RES	Number of lipids
ceramide (Cer)	sphingolipids (SP)	1.19<br>*1.72E-01*	2.48<br>*6.79E-07*	1.99<br>*2.28E-02*	5.95<br>*4.47E-02*	3.02<br>*4.01E-03*	1.04<br>*7.59E-01*	22
cardiolipin (CL)	glycerophospholipids (GP)	-1.06<br>*6.14E-01*	1.22<br>*1.90E-01*	-1.10<br>*6.02E-01*	3.26<br>*1.41E-02*	1.78<br>*5.51E-02*	1.13<br>*2.80E-01*	3
diacylglycerol (DG)	glycerolipids (GL)	1.14<br>*8.54E-02*	1.24<br>*1.82E-03*	1.51<br>*3.42E-02*	4.16<br>*2.88E-01*	1.55<br>*4.67E-02*	1.01<br>*8.59E-01*	32
glucosylceramide (GlcCer)	sphingolipids (SP)	-1.08<br>*3.11E-01*	2.63<br>*2.89E-06*	1.04<br>*7.50E-01*	3.76<br>*6.33E-03*	2.70<br>*1.20E-04*	-1.14<br>*1.71E-01*	7
phosphatidic acid (PA)	glycerophospholipids (GP)	1.02<br>*4.81E-01*	1.03<br>*1.99E-01*	-1.01<br>*6.23E-01*	1.21<br>*6.49E-02*	1.04<br>*2.06E-01*	-1.03<br>*2.40E-01*	30
phosphatidylcholine (PC)	glycerophospholipids (GP)	1.02<br>*3.77E-01*	1.11<br>*2.71E-03*	1.04<br>*6.52E-02*	1.18<br>*2.49E-02*	1.08<br>*1.65E-02*	1.04<br>*1.64E-01*	279
phosphatidylethanolamine (PE)	glycerophospholipids (GP)	-1.11<br>*8.56E-02*	-1.22<br>*1.49E-03*	-1.20<br>*2.24E-03*	-1.22<br>*2.35E-03*	-1.27<br>*4.43E-03*	-1.06<br>*2.19E-01*	58
phosphatidylglycerol (PG)	glycerophospholipids (GP)	-1.06<br>*1.97E-01*	-1.18<br>*6.64E-04*	-1.12<br>*7.79E-03*	-1.25<br>*2.14E-04*	-1.26<br>*6.38E-04*	-1.03<br>*3.99E-01*	45
phosphatidylinositol (PI)	glycerophospholipids (GP)	-1.04<br>*7.57E-01*	1.38<br>*3.15E-02*	-1.28<br>*1.41E-01*	1.75<br>*1.76E-02*	1.35<br>*6.63E-02*	-1.22<br>*2.62E-01*	42
phosphatidylserine (PS)	glycerophospholipids (GP)	-1.02<br>*6.15E-01*	1.15<br>*2.21E-02*	-1.01<br>*7.95E-01*	1.03<br>*4.18E-01*	1.07<br>*2.47E-01*	-1.01<br>*8.52E-01*	91
sphingomyelin (SM)	sphingolipids (SP)	-1.02<br>*4.46E-01*	-1.32<br>*3.35E-04*	-1.04<br>*3.81E-01*	-1.16<br>*1.55E-02*	-1.35<br>*8.36E-05*	-1.11<br>*1.19E-02*	20
triglyceride (TG)	glycerolipids (GL)	-1.02<br>*4.60E-01*	-1.24<br>*5.08E-04*	-1.03<br>*4.57E-01*	-1.14<br>*8.99E-03*	-1.28<br>*6.66E-05*	-1.09<br>*8.34E-03*	36
